# The Italian forest carbon sink: historical evolution and future projections under alternative harvest scenarios

**DOI:** 10.1186/s13021-026-00486-6

**Published:** 2026-07-18

**Authors:** Roberto Pilli, Emil Cienciala, Kevin Black

**Affiliations:** 1 Independent Consultant, 35133 Padua, Italy; 2 IFER – Institute of Forest Ecosystem Research, 254 01 Jilove U Prahy, Czech Republic; 3 FERS Ltd, Kilberry, Navan, Co Meath Ireland

**Keywords:** Carbon accounting, Forest management, Felling rates, Afforestation, Natural disturbances

## Abstract

**Background:**

In 2023, forests offset approximately 9% of total GHG emissions in the EU-27, with Italy accounting for about 20% of these offsets. Historical felling rates, in Italy, are highly uncertain, with a ± 50% confidence interval. In this study, we used a forest carbon inventory model, scaled at regional level, to assess the overall impact of the harvest uncertainty on the Italian forest carbon (C) sink. The analysis included most recent information from the literature and an ancillary assessment of the harvested wood product mitigation potential. Based on this assessment, we estimate the evolution of the forest C sink to 2070, under different harvest scenarios defined by an increasing felling rate relative to the net annual increment estimated by the model on forest area available for wood supply.

**Results:**

Comparison of our results with the Italian GHGI data shows an overall good fit with the living biomass sink, but significantly higher removals by the dead organic matter pool. For the historical period, the modelled total C sink increases from -40 Tg CO_2_ yr^−1^ in 2005 to -55 Tg CO_2_ yr^−1^ in 2023.

The future forest C sink varied between -76 Tg CO_2_ yr^−1^ and -16 Tg CO_2_ yr^−1^ by 2070, corresponding to the lowest (0.10) and highest (0.90) felling to net annual increment ratios, respectively. Our analysis confirms that the contribution of harvested wood products to the total C sink is negligible, as the Italian forest sector predominantly channels timber extraction into energy production.

**Conclusions:**

Between 2005 and 2023, the impact of harvest uncertainty on the total forest C sink averaged 11 Tg CO_2_ yr^−1^, representing about ± 23% of the C sink associated with the average harvest reported in the literature for the same period.

By simulating the theoretical forest C sink dynamics under different felling rates, and by excluding any possible impacts of climate change on current growth conditions, our study provides a long-term assessment of wood production potential versus carbon storage capacity. This bottom-up modelling framework aligns with the New EU Forest Strategy and can serve as a model for other European countries.

**Supplementary Information:**

The online version contains supplementary material available at https://doi.org/10.1186/s13021-026-00486-6.

## Background

In 2023, forests offset about 9% of total GHG emissions reported by EU 27 [1]. Whilst most of Northern and Central European countries report a decreasing C sink in recent years, Italy contributes the largest net CO_2_ forest removals, when compared to other EU Member States. In 2023, the EU 2025 National Inventory Document [1] reports that about 20% of the total net CO_2_ removals reported by EU can be attributed to Italian forest categories, including Forest Land remaining Forest Land (FL-FL) and Land Converted to Forest Land (L-FL). Between 1990 and 2023, the national C sink reported for the categories FL-FL and L-FL increased by 176% and 374%, respectively, with L-FL providing about 25% of the C sink attributed to total FL in 2023 [2].

Some studies have assessed the carbon sequestration capacity of the Italian forests at local and national level, including impacts of climate change and natural disturbance events [3–5]. However, the increasing role of the Italian forests within the EU LULUCF sector highlights the need for new research and development of refined tools to assess both the historical and the future evolution of the forest C sink in Italy. In addition, a re-assessment of the historical evolution of the Italian forest carbon sink is required because of availability of new data sources. These data include the results from the third Italian National Forest Inventory (hereafter, INFC 2015 [6]), a new in-depth analysis of historical harvest rates based on remote sensing data [7], a detailed assessment of the cascade effects from the exceptional windstorm in autumn 2018 and new data on forest area expansion [2].

As highlighted by previous studies, estimation of the evolution of the Italian forest C sink is highly uncertain, because of the lack of accurate data on the amount of harvest [3, 5].

Together with the possible impact of climate change and natural disturbances, which are not under direct human control, and of age structure, which is mostly determined by past management strategies, harvest is the main driver affecting the short to medium term evolution of the forest C sink.

Despite the availability of harvest data from the INFC 2015 [6] and the ongoing efforts to assess the recent evolution of the amount of harvest [8], the determination of the historical harvest rate remains challenging. A more recent attempt to assess the historical evolution of the amount of fellings over the period 2000 and 2023 suggests that total fellings—including both total removals and logging residues—ranged from a peak of 16.4 million m^3^ in 2006 to a low of 10.4 million m^3^ in 2014 [7]. The same study, however, highlights that, despite efforts to integrate and use different data sources, the uncertainty of these values is quite large (± 50%). Other studies suggest that harvest statistics in Italy are generally underestimated, also because harvest from the informal market are not included in national statistics, and therefore, the likely harvest is most probably higher than current estimates reported by the GHGI [9–11]. The first objective of this study is to assess how different historical harvest levels, derived from the literature, impact the Italian forest C sink by simulating its evolution over the period 2005—2023. To achieve this, we integrated a forest carbon inventory model (Carbon Budget Model [12]) with most recent information from the literature, specifically between 2005 (corresponding to the second Italian National Forest Inventory, hereafter INFC 2005 [13]) and 2023 (based on the Italian National Inventory Document [2]). We also included ancillary information published by FAOSTAT [14] to assess the HWP mitigation potential.

Considering the increasing role of the LULUCF sector in the national [15] and EU [16] legislation and large uncertainties in the future harvest rate, the second objective of this study is to provide an assessment of the future evolution of the forest C sink, under different harvest scenarios. In the short to medium term, forest management activities, and particularly harvest intensity, is, by far, the main driver shaping the forest carbon sink, provided that climate change impacts on growth conditions are excluded [17]. To ensure a long-term, sustainable management regime, however, the amount of fellings must be compared with the evolution of the net annual increment (NAI [18]). The ratio between fellings and NAI, also known as felling rate, is a well-established parameter to assess the intensity and impact of forest management activities, not only on the future harvest supply chain, but also on other ecosystem services [18]. Given the specific characteristics of the Italian forests — where approximately 66% of the area is privately owned and highly fragmented, mostly located in mountain regions, and 54% stands are on slopes steeper than 40% — consideration of the forest area available for wood supply (FAWS) and not available for wood supply (FNAWS) is crucial. The FNAWS, which covers about 10% of the total forest area, is generally forest land where some legal, economic, or environmental restrictions prevent management activities such as wood harvesting [6, 19]. Therefore, as part of the second component of this study, we assessed the evolution of the Italian forest C sink from 2024 to 2070, under different harvest scenarios, defined by an increasing felling rate assessed on the FAWS. This is an essential requisite to support a long-term, strategic assessment of forest resources available at the national level.

## Methods

### Modelling framework

The study includes two main steps, summarized in Table 1. The first step aims to assess the historical evolution of the Italian forest C sink, as determined by the combined effects of harvest, afforestation and natural disturbances occurred within the period 2005 – 2023, including a sensitivity analysis of the impact of the minimum and maximum amount of felling rates inferred by literature within the same historical period. The second step aims to estimate the evolution of forest C sink to 2070, under nine harvest scenarios, corresponding to various felling rates, which increase from 10 to 90% of the ratio between felling and NAI, determined for FAWS areas only. These wide-ranging harvest scenarios test the extremes of different policy pathways: a progressive increase in future harvest rates, as projected by the Italian National Forest Accounting Plan [20], versus a stabilization or reduction of current felling levels, which could better comply with the stringent targets set by EU climate legislation [21]. All of the analysis was carried out using the Carbon Budget Model (CBM), scaled at NUTS 2 level, and accompanied by a complementary assessment of the HWP mitigation potential, based on the IPCC Tier 2 method [22].

**Table 1:** Main assumptions applied within the historical period 2005 – 2023 and the projection period 2024 – 2070. Harvest column reports the average harvest (i.e., total merchantable removals of roundwood and a share of non-merchantable wood residues) estimated within the historical period, according to data reported by [7]

Period/Step	Harvest	Natural disturbances	Afforestation, deforestation, HWP
Historical period 2005 – 2023	As determined by [7], with three harvest levels: • *AvgHistHrv*: ≈ 11 Mm^3^ yr^−1^ o.b • *MaxHistHrv*: ≈ 16 Mm^3^ yr^−1^o.b • *MinHistHrv*: ≈ 6 Mm^3^ yr^−1^ o.b	• Wildfires (2005 – 2023) • Windstorms (2018) • Bark beetle (2020 – 2023) • Salvage logging (2019–2023)	• ARD: as determined by INFC [6, 13] and GHGI [2] • HWP: based on FAOSTAT data [14]
Projection period 2024—2070	9 harvest scenarios corresponding to different felling rates, from 10 to 90% of NAI assessed on FAWS	Constant average fire level, equal to the average area affected by wildfire within the historical period, excluding further disturbance events due to windstorms and insects’ outbreaks	• No land use change • HWP: varying with each harvest scenario, with steady-state ancillary conditions determined on the historical period

### The Carbon Budget Model

The Carbon Budget Model (CBM-CFS3, hereafter CBM) is an inventory-based, yield-data driven model that simulates, with annual time steps, the stand- and landscape-level C dynamics of above- and below-ground biomass, dead organic matter, and mineral soil pools [12]. Each forest stand is characterized by age, area and various classifiers that provide administrative and ecological information, that is linked to appropriate growth curves, and other parameters defining, for example, the forest composition and management strategy. Species-specific, stand-level equations convert the merchantable volume reported by growth curves into various aboveground biomass components [3, 23]. The model uses an initialization process to estimate the size of all DOM pools at the start of the simulation and then links DOM dynamics to biomass dynamics. Biomass transfer is driven by a set of turnover rates, litterfall rates, and temperature-dependent decay rates.

During the annual model run, eligible stands are selected based on classifiers and subjected to natural or anthropogenic disturbances; their impacts are determined by a ‘disturbance matrix’ that defines the proportions of carbon transferred between pools, harvested, or released to the atmosphere [24]. Concurrently, merchantable volumes from growth curves are converted into aboveground biomass components using species-specific allometric equations.

For this study we used the EU Archive Index Database (EU AIDB) made available by the Joint Research Centre of the European Commission [25], which was further adapted to the Italian case study, according to a new model assessment, described within the following sections. The model was run both using the CBM User interface and a specific Python script made available by the Canadian Forest Sector (courtesy of Scott Morken, from the Canadian Forest Sector).

### CBM parametrization

In 2012, Italian forests were taken as prototype for testing the application of the CBM within EU Member States [3]. In subsequent years, the same model has been repeatedly applied to Italy and other European countries (e.g., [5, 26, 27]), but most of the input data and methodological assumptions applied for Italy have continued to be based on the first modelling assessment, as described in [3]. For this study, all input data, including the historical amount of harvest, were reassessed and, when possible, updated using new information made available by literature and a critical assessment of previous methodological assumptions. An overview of the modelling parametrization process is provided in Supplementary Materials (Appendix A).

#### Area parametrization

Input data used for defining the forest area at the beginning of the model run were derived from INFC 2005 data [13]. To match the spatial scale of the INFC data and enhance the granularity of model outputs, all input data were scaled to the NUTS2 level wherever possible (see Supplementary Materials, Appendix A). We preliminarily distinguished the following categories, which were treated separately (Table 2): even-aged high forests, uneven-aged high forests, coppices, plantations, special management systems. By combining the information on management types with management systems reported in Table 2, we defined the following classes: even-aged high forests (HU), uneven-aged high forests (HU), simple coppices (CS), coppices with standards (CM), coppices with high forests (CH) and special management types (SE).

**Table 2:** Distribution of the forest area reported by INFC 2005 between different management types and systems considered by CBM

INFC management type	INFC management system	Area (kha)	CBM classes	Assumptions/Notes
**High forests**	Even-aged	1,485	HE	Including original even-aged high forests (1,270 kha) + coppices in transition to high forests (151 kha) + even-aged high forests not classified by age classes (238 kha)
	Uneven-aged	2,560	HU	Including original uneven-aged high forests (1,156 kha) + Irregular high forests (493 kha) + high forests not defined for management system (889 kha) + 23 kha not classified by age classes
	**TOTAL High forests**	**4,045**		
**Coppices**	Simple	872	CS	
	With standards	2,408	CM	
	With high-forests	383	CH	
	**TOTAL Coppices**	**3,663**		
**Special**	Uneven-aged	118	SE	No age class distribution reported by INFC
**Not classified**	-	757	HE/HU/C	Proportionally distributed between HE-HU-C
**Plantations**	Even-aged	122	HE	Each age class was proportionally distributed between Poplar Plantations, Broadleaves Plantations and Conifers Plantations
**Temporary unstocked area**	-	54	HE/C	Assigned to age class 0 under even-aged high forests and coppices
**Total forest area**		**8,759**		

The total forest area was allocated over 21 administrative units (mostly defined at NUTS 2 level) and 20 forest types (FT) based on the species composition reported by INFC 2005 (see Supplementary Material, Appendix A). Forest types were grouped by coniferous and broadleaves species groups, and between FAWS and FNAWS. FNWAS is used as proxy the area not available for wood supply reported by INFC 2005 (Table 6.5, [13]). To this purpose, we first defined the share of forest area not available for wood supply for each region and FT, and then we proportionally assigned FNAWS area in the data set. The final distribution of the forest area between FAWS and FNAWS –constant since 2024 onward—is reported in Appendix A.

The forest area associated with even-aged forest management systems (including coppices) was initially distributed between 14 age classes with a 10-year bin width, based on ancillary information reported by INFC 2005. The uneven-aged forest area was attributed to a single age class according to specific methodological assumptions described below.

#### Growth curves

Growth curves define, for each species, the gross merchantable volume per ha and age class, i.e., the volume under bark of the main stem, as expected in the absence of disturbances and management activities [23]. Since the volume data reported for growth and standing volume is used both for assessing the standing volume at the beginning of the model run (time step 0) and the periodic annual increment applied during the simulation stage, we used two different growth curves libraries [3]. The initialization yield tables (YT) were applied to assess the standing stock at the beginning of the model run; the current YTs were applied during the model run, to assess the expected annual increment in the absence of disturbances or management activities, i.e., the periodic increment, excluding mortality due to stand dynamic (self-thinnings), which is computed through separate model’s parameters [3, 12].

INFC data report the stem volume over bark including large branches with a minimum diameter > 5 cm and the corresponding current annual increment, which is derived by the annual increment of the stem radius, determined using a core sampling technique [6, 13]. For this reason, increment data reported by INFC represent the gross annual volume increment, equal to net annual increment plus annual natural losses [28]. Input data were preliminarily harmonized to meet the CBM model requirements (i.e., volume and increment definition) by applying different correction factors. To exclude bark and large branches from volume data (at least for coniferous species) and trees with Dbh ≤ 9.0 cm, we applied an overall correction factor (CF) equal to 0.96 for coniferous species and 0.89 for broadleaves (Table 3). To exclude annual natural losses from the current annual increment values reported by INFC, and other components such as large branches and small trees, we applied a CF equal to 0.76 for broadleaves species and 0.86 for coniferous species. Correction factors applied to increment were inferred by comparing INFC increment data with comparable data harmonized according to a common increment definition, excluding trees with a Dbh ≤ 7.5 cm, branches for coniferous species, and annual natural losses. All this information was derived from the data reported by [30] and from the ancillary information used by these authors. After a preliminary model assessment, we concluded that these corrections factor out most of the differences between INFC data and model’s definitions.

**Table 3:** Comparison between the volume and increment components considered by INFC, the harmonized increment definition reported by Avitabile et al. [29] and the volume and increment components as defined by CBM. Correction factors applied to INFC volume and increment data (highlighted by bold values) are derived by the ratio between original INFC data and harmonized increment data, distinguished between coniferous and broadleaves species

	INFC definition	Harmonized definition Avitabile et al. 2024	CBM definition	Correction Factor
Min Dbh	Dbh > 4.5 cm	Dbh ≥ 7.5	Dbh ≥ 9.0	
Stem components	Stem above stump height to top diameter of 5 cm + large branches with minimum diameter 5 cm	Stem over-bark from stump height to top diameter of 7 cm + large branches with minimum diameter of 7 cm for broadleaves	Volume and NAI for merchantable stem under bark, excl. branches	
Annual Nat Losses	Included	Excluded	Excluded	
GAI conif	5.8 m^3^ ha^−1^ yr^−1^	5.6 m^3^ ha^−1^ yr^−1^	5.6/5.8 = **0.96**	Vol conif
GAI broad	3.7 m^3^ ha^−1^ yr^−1^	3.3 m^3^ ha^−1^ yr^−1^	3.3/3.7 = **0.89**	Vol broad
NAI conif	Not available	5.0 m^3^ ha^−1^ yr^−1^	5.0/5.8 = **0.86**	GAI conif
NAI broad	Not available	2.8 m^3^ ha^−1^ yr^−1^	2.8/3.7 = **0.76**	GAI broad

**Fig. 1:**
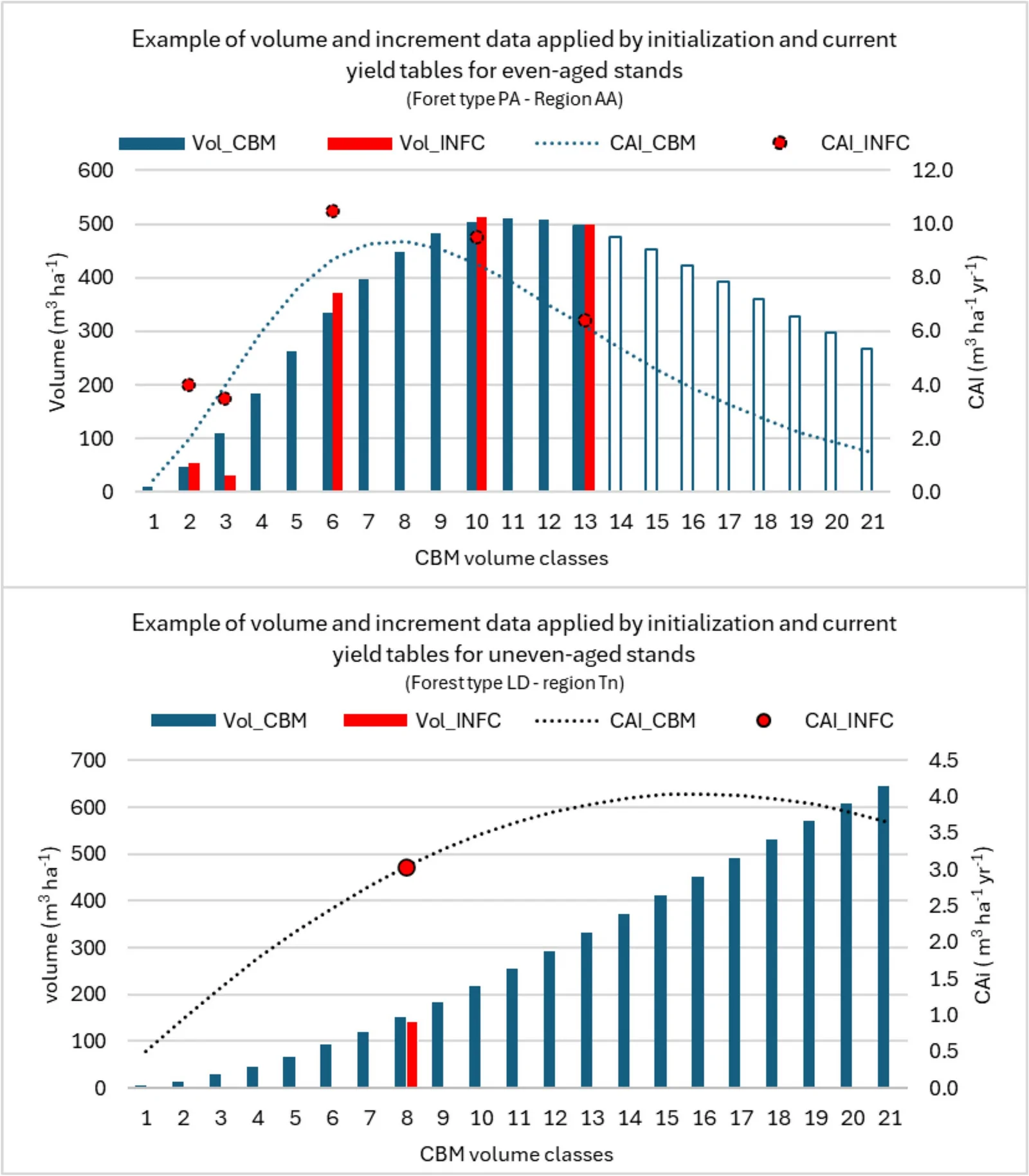
The upper panel reports an example of volume (Vol, plotted on the left y-axis) and increment (referred to Current Annual Increment, plotted on the right y-axis) data applied by initialization and current yield tables for even-aged stands. Data used by CBM (Vol_CBM and CAI_CBM) are also compared with the corresponding volume and increment data reported by INFC 2005. Please note that INFC volume data, used for the initialization stage, only refers to age classes < 13 (i.e. age classes > 13 are not applied by model because missing within the initialization stage). The lower plot, referred to uneven-aged stands, reports an example of cumulated volume per ha (plotted on the left y-axis) derived from the current annual increment values (plotted on the right y-axis) associated to each volume class (reported on the x-axis). Red points highlight the volume and increment values derived by INFC 2015 input data (preliminarily corrected), for uneven-aged forest stands within the same forest type and region

Growth curves applied to even-aged stands were derived from volume and increment data reported by INFC 2005 for even-aged high forests, coppices, and plantations [13]. Volume data were used to derive the initialization YT and increment data were used for deriving the current YT applied during the model run (see the example reported in Fig. 1). In both cases, we started by using, if available, volume and increment data distributed by forest type, management type (distinguishing high forests and coppices), regions and age classes. Additional details are reported in Supplementary Material, Appendix B.

To represent the growth dynamics of uneven-aged forests, including coppices, in the CBM modelling framework—which operates on an age-based growth structure—a specific methodological approach was implemented based on the "virtual age-class" concept [3]. Each volume class was considered as equivalent to an age class and the area assigned to each uneven-aged FT and region, was allocated within a unique volume class. Growth curves applied to uneven-aged stands were derived from INFC 2015 plot level data [31]. In this case, current annual increment values measured by INFC 2015 were used to derive, for each FT and region, species-specific uneven-aged growth curves including 21 volume classes, with the volume of each class inferred from current annual increment reported by INFC data (see the example reported in Fig. 1 and further details reported in Supplementary Material, Appendix B). Classification of productivity classes for forest types was not feasible due to the lack of site-specific productivity indicators in the INFC 2015 inventory. To correctly simulate the initial standing volume at the beginning of the model run (time step 0), we assigned the standing volume to each uneven-aged stand volume-class based on the standing volume reported by INFC 2005. To this purpose, for each FT and region, each uneven-aged stand reported within the CBM inventory input data was first associated to the volume reported by the corresponding uneven-aged growth curve, and then to the equivalent volume-class, assumed, within the CBM modelling framework, as a "virtual" age class (see the example reported on Fig. 1). Therefore, the initial age classes assigned to uneven-aged stands within the CBM inventory input data vary by region and FT, depending on the corresponding growth curve. Unlike even-aged systems, which follow a clear trajectory toward a rotation age, the growth of uneven-aged stands was parameterized to achieve a long-term “near steady-state”, where harvest and growth, including recruitment rates, are equivalent. This was achieved by calibrating the yield curves so that the NAI remained consistent using the regional averages inferred by INFC 2015 plot level data. This effectively simulates the continuous recruitment and selective harvesting processes typical of irregular forest structures. The same assumptions were also applied to the special management type assigned to the forest types CS and QS, where no age class distribution is reported by INFC data.

#### Allometric equations

To improve the consistency of the model output with the estimates reported by INFC 2015, the allometric equations used within previous studies were further revised. Original allometric equations for Italy, as reported by the EU AIDB [25], were selected by comparing the average volume reported by INFC 2005, for each administrative region and forest type, with corresponding volume derived by a database including 191 stand-level equations provided by Boudewyn et al. [23]. Selection of final allometric equations was based on the goodness of fit for each equation by comparison with the biomass wood density reported in the literature (derived by the Italian National Inventory Report) and with the basic wood density inferred by a preliminary model output, including various allometric equations, where the wood density inferred by model (*WD*_*cbm*_) was estimated as:

1 $${WD}_{cbm}=\frac{{Merch\_bio}_{cbm}}{{Merch\_vol}_{cbm}}$$

where, *Merch_bio*_*cbm*_ is the merchantable C biomass reported by the model output, and *Merch_vol*_*cbm*_ is the corresponding merchantable volume estimated by the allometric equation associated to each FT.

Once the new set of allometric equations for a limited number of regional observations (n = 20 forest types) was selected, the model performance was evaluated using multiple statistical indicators, including the Mean Absolute Error (MAE = 0.04 Mg m^3^) and normalized RMSE (nRMSE = 10%) to assess the precision and bias of the estimates. The models showed a satisfactory performance with a percent bias (PBIAS) of -19%, indicating a moderate tendency to underestimate the wood density, compared to data provided by literature, however, we notice that, for the main species (i.e., AA, FS, PA, QC) the wood density inferred from the model output is well aligned with the values reported by literature (see Supplementary Material, Appendix C).

A detailed list of the new association between original allometric equations [25] and Italian FTs is reported as Supplementary Material (Appendix C).

#### Calibration of dead organic matter pools

The CBM simulates DOM fluxes associated with biomass turnover, harvest and other disturbances using a 21 C pool flow model which represent the 5 IPCC pools (above and below ground biomass, litter, deadwood and soils [12]).

The original EU AIDB includes a set of predetermined biomass annual turnover and decay rates, mostly based on the default parameters provided by the Canadian Forest Sector. For the present study, some of these parameters were modified using more recent information reported in the literature followed by a calibration of the model outputs (see Table 4). In particular, the default biomass annual turnover rates were generally increased, based on specific Italian studies [32], or other temperate and Mediterranean forests [33, 35].

**Table 4:** Main biomass and DOM turnover and decay rates modified within the present study (AIDB updates), further compared with the default values reported within the EU AIDB. The last column and notes report detailed information on the data sources applied for calibrating each parameter

	Parameters	EU AIDB	AIDB updates	Source
BIOMASS TURNOVER RATES	AverageAge	Varies	90 yrs	Calibr. in present study
	StemAnnualTurnOverRate	0.003	0.0048	[i]
	HardwoodBranchTurnOverRate	0.04	0.0425	[ii]
	SoftwoodBranchTurnOverRate	0.04	0.0425	[ii]
	HardwoodFoliageFallRate	0.95	0.95	-
	SoftwoodFoliageFallRate	0.11	0.11	-
	HardwoodCoarseRootsTurnOverRate	0.02	0.38	[iii]
	SoftwoodCoarseRootsTurnOverRate	0.02	0.38	[iii]
	HardwoodFineRootsTurnOverRate	0.64	1.11	[iv]
	SoftwoodwoodFineRootsTurnOverRate	0.64	1.11	[iv]
DOM TURNOVER & DECAY RATES	HardwoodStemSnagToDOM (to Medium)	0.032	0.0009 – 0.3239	[v]
	SoftwoodStemSnagToDOM (to Medium)	0.032	0.0009 – 0.3239	[v]
	HardwoodBranchSnagToDOM (to AG Fast)	0.10	0.001 – 0.25	[vi]
	SoftwoodBranchSnagToDOM (to AG Fast)	0.10	0.001 – 0.25	[vi]
	Medium decay rate	0.0374	0.0350	[vii]
	BG slow decay rate	0.0033	0.0066	[vii]
	BG very fast Q 10	1.0	2.65	[viii]
	Medium Q 10	2.0	2.0	-
	Medium Proportion to atmosphere	0.83	0.90	[ix]

[i]: average volume mortality rate determined at country level excluding Douglas Fir (see [32] Table 1)

[ii]: mean coarse woody debris, as reported by [33] on Table 5

[iii]: average coarse root decomposition rate suggested for middle latitude areas [34]

[iv]: revised fine-roots turnover rates suggested for *Fagus sylvatica* and *Picea abies*, applied to broadleaves and coniferous species, respectively [35]

[v] Climate-dependent decay rates derived from data compilation of decay constants of Fagus sylvatica and other deciduous trees occurring in temperate climates, as reported by [36]

[vi] Climate-dependent CBM decay rates modified proportionally to the decay rates attributed to Stem Snags

[vii] Calibration on present study according to parameters reported by [37]

[viii] Increase default Q10 according to [38]

[xi] Maximum proportion to atmosphere (0.70 – 0.90) suggested by [37] for medium soil pool

**Table 5:** The table reports the correlation (r) between the data reported by FAOSTAT [14], within the period 2010—2020, for (i) the production of industrial roundwood coniferous species (IRW_C_) vs sawn wood coniferous; (ii) the production of industrial roundwood non-coniferous species (IRW_NC_) vs sawn wood non-coniferous; (iii) IRW_C_ and IRW_NC_ vs wood based panels; (iv) IRW_C_ and IRW_NC_ vs paper & paperboard and wood pulp. The last column reports the methodological assumption (“constant value” or “proportional variation”) applied within the period 2024 – 2070, to model the evolution of each commodity and the corresponding average constant value (if considered)

Commodity	Correlation		Assumption
	with IRW_C_	with IRW_NC_	(avg. 2010–2020)
**Sanwood coniferous**	-0.85	-	constant avg. (798,925 m^3^ yr^−1^)
**Sanwood non-coniferous**	-	0.89	Proportinal variation with IRW_NC_
**Wood-based panels**	0.43	0.04	Proportinal variation with IRW_C_
**Paper and paperboard**	0.04	0.11	constant avg. (8,855,334 Mg yr^−1^)
**Wood pulp**	-0.72	0.45	Proportinal variation with IRW_NC_

DOM turnover rates were also revised and modified using a temperature and precipitation gradient factor, derived by a large data compilation of decay constants of beech (*Fagus sylvatica*) and other deciduous trees occurring in temperate climates as reported by [36]. Based on these parameters, we defined a general precipitation-dependent turnover function, used to estimate the mean annual stem turnover rate (*k*) associated to different climatic units, defined according to the total annual precipitation (*P*) associated to each Climatic Unit. The relationship was modeled using a logarithmic regression in the form:

2 $$k=0.2393*\mathrm{ln}\left(P\right)-1.4589$$

The parameters were estimated by linearizing the independent variable and the regression yielded a high level of significance for the b coefficient (p < 0.05, 95% CI: 0.0151, 0.4634), with an R^2^ of 0.91.The advantage of this equation is to clearly link the decay rate with environmental conditions, and, in particular, with the precipitation gradient. Indeed, various studies highlight that the decay rate is largely influenced by soil moisture [39]. Climatic conditions associated to each forest unit were defined by a specific classifier, named Climatic Unit (CLU), already described in [40]. Default CBM decay rates associated with snag branches were also proportionally modified, according to the relative variation attributed to the same parameters for stem snags. For stem decay rates < 0.001, branches decay rate was aligned with stem decay rate, for stem decay rates > 0.001, branches’ decay rate was reduced to 78% of stem decay rate. Other parameters were modified according to the methodological assumptions reported in Table 4.

#### Disturbance events

Disturbance events are divided into three main categories: harvesting, natural disturbances, and land use change activities. Harvesting includes ordinary silvicultural activities and salvage logging after natural disturbances. Natural disturbances, in case of Italy, include wildfires, windstorms, and biotic disturbance events, such as bark beetle outbreaks. Land use change activities include afforestation (AR) and deforestation (D).

Annual removals were inferred from a specific study based on the integration of National Forest Inventory data with yearly time series derived by remote sensing data [7]. Starting from the total amount of fellings estimated by this study at national and regional level, we inferred the total amount of removals which were used to define the input data required by model, assuming a constant correction factor equal to 0.85, to account for logging residues, which moves from living biomass to DOM pools (see Supplementary Materials, Appendix D).

Total removals scaled at regional level were further distributed between various FT, management type, management systems and disturbance events, according to specific methodological assumptions, reported as Supplementary Material (Appendix D), and further converted from volume to tons of C using species specific wood density values, estimated, for each FT, as the ratio between biomass and volume reported by INFC 2005 for merchantable woody biomass [13], with a constant carbon fraction equals to 0.5.

In autumn 2018, an extra-tropical windstorm, named Vaia, blew down about 9.5 million m^3^ of merchantable timber in Northern Italy [5]. The windstorm predominantly affected pure Norway spruce stands, and mixed Norway spruce – Silver fir and Norway spruce – European beech stands. The effect of storm Vaia was simulated through four different disturbance events, with an increasing share of living biomass moving to DOM pools (see Supplementary Material, Appendix E) and further allocation to different administrative units and FTs according to the general assumptions already suggested by [5]. For the present study, the area and minimum merchantable C stock affected by each disturbance class were recalibrated to match the total area and volume affected by Vaia within each region using the latest data reported in the literature (see Supplementary Material, Appendix E).

Salvage logging carried out from 2019 to 2023 was simulated with a specific silvicultural treatment (disturbance event) by moving 90% of the windblown merchantable standing dead-biomass pool (snag stems, following storm Vaia) to the harvested products pool. The affected windthrow area was calibrated to match the amount of salvage logging reported in the literature for each region and FT over the simulation period 2019–2020. For the three regions most affected by this event, we also assumed that after salvage logging, 30% (for Veneto region) and 15% (for Alto Adige and Trento autonomous provinces) of the forest area originally covered by spruce and fir stands, were replaced by beech forest stands.

Recent studies (e.g., [41, [42]) highlight that, since 2020, the regions affected by Vaia have also experienced a severe bark beetle outbreak. This infestation peaked over the period 2021 and 2023 and was driven by the large volumes of deadwood and favorable climatic conditions. The overall volume affected by this bark beetle infestation was assumed to be the same as the areas affected by storm Vaia. No additional windstorm or bark beetle disturbance event was applied for the simulation period 2024 – 2070.

**Fig. 2:**
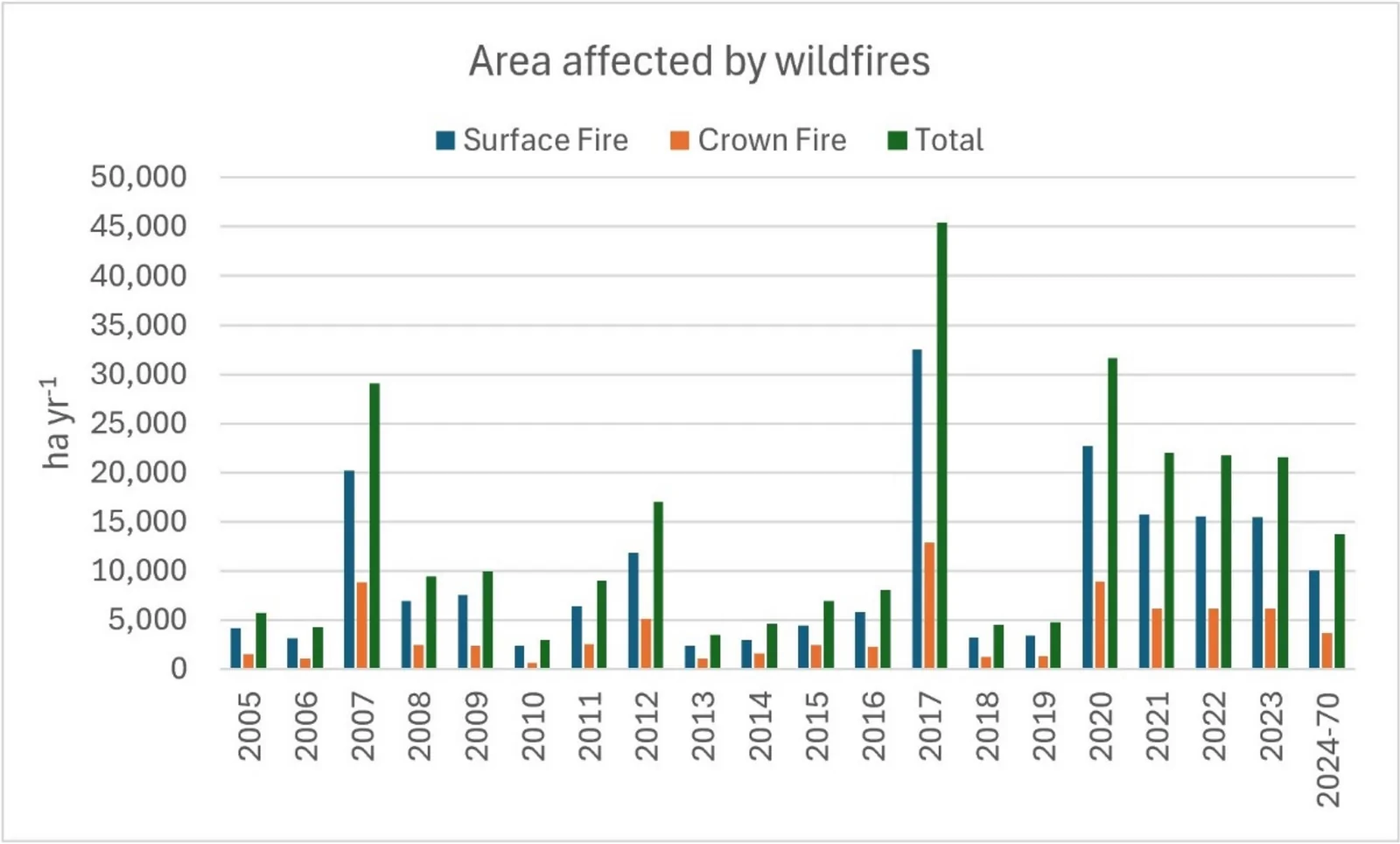
Total area affected by wildfires within the historical period 2005 – 2023 and the following projection period (2024 – 2070, assumed as constant), grouped by surface fires and crown fires disturbance events

Wildfires are the main disturbance events affecting Italian forests, mostly within the Mediterranean and Pre-Alpine regions. The forest area affected by wildfire was estimated from the data reported, for each administrative region, by the Forest Information System for Europe [43]. This area, which included the land use categories Other Wooded Land and Grasslands, was scaled against the total area affected by fire, as reported by the Italian Common Reporting Tables (CRT) for the category Forest Land [44]. In this way, we preliminarily excluded wildfires occurring on non-forest lands. Because FISE does not report data after 2015, since 2016 onward, we proportionally distributed between various administrative regions the burned area reported by the UNFCCC CRT tables against the average burned area derived, for each region, for the period 2010–2015. The total forest area affected by wildfires within the historical period 2005 – 2023 was further distributed between two disturbance events, representing crown fires and surface fires, as defined by INFC 2005 (Fig. 2). For this purpose, we directly inferred from INFC 2005 the proportion of area affected by crown fires and surface fires within each administrative region, and we assumed as constant this proportion within the entire simulation period. Crown fires were associated to a stand-replacing disturbance event affecting 80% of living biomass pool, including 5% of salvage logging on merchantable standing stock. Surface fires were associated to a disturbance event, mostly affecting dead wood and litter pools and only 20% of merchantable living biomass. Within each region, the forest area assigned to crown fires and surface fires was finally distributed, for each time step, proportionally to the forest area available and not available for wood supply, grouped by broadleaves and coniferous FTs, as inferred from the inventory database.

The forest area affected by wildfires over the projection period 2024 – 2070, was estimated as the sum of the average area affected by wildfires, at regional level, within the entire historical period, excluding, for each region data series, the extreme outliers (burned area) defined as those exceeding three times the interquartile range from the median area affected by wildfires within each region, within the historical period [45]. The resulting forest area affected by wildfires, equal to 13,758 ha yr^−1^ at national level, was proportionally distributed between FAWS and FNAWS, coniferous and broadleaves species groups, and crown fires (about 27% of the burned area, within the historical period) and surface fires (about 73% of the burned area), according to the average proportion assigned to each group within each region, on the historical period 2005–2023.

The total afforestation and deforestation rates between 2005 and 2015 were determined by the difference between the forest area reported, for each region and FT, by INFC 2015 (${A}_{reg, FT}^{2015}$) and the corresponding forest area reported, for the same categories, by INFC 2005 (${A}_{reg, FT}^{2005}$). INFC 2015 data were preliminarily harmonized to INFC 2005, and the area not classified by FT was proportionally distributed between the remaining categories. When, for each region and FT, the difference between ${A}_{reg, FT}^{2015}$ and ${A}_{reg, FT}^{2005}$ was positive, the area was assumed as afforested land and assigned to the corresponding disturbance event; when the difference was negative, the area was assumed as deforested land. The final dataset was proportionally distributed between FAWS and FNAWS, according to the relative share assigned to each category within the INFC 2005 data. The annual rate of afforestation (40.4 kha yr^−1^) and deforestation (11 kha yr^−1^) occurred between 2005 and 2015 was determined by dividing by 11 years the area assigned to AR and D, respectively. The total net difference between AR and D, equal to 322 kha, is in line with the absolute difference between the total forest area reported by INFC 2005 (8,759 kha) and INFC 2015 (9,085 kha). Between 2016 and 2023 the net annual afforestation rate (202.4 kha yr^−1^, including the impact of deforestation over the same period) was inferred from the data reported by the latest Italian National Inventory Document [2]. The effective afforestation and deforestation rates applied within the model run are generally slightly lower than the theoretical ones. No land use change was applied since 2024 onward.

Since our modelling framework starts in 2005, we did not consider, for the land use category L-FL (i.e., afforestation) the conventional 20 years transition period as defined by IPCC, but we directly reported the C sink attributed to each pool on total forest land. We applied an annual area change approach which takes the annual variation on the total forest area into account, as determined by afforestation and deforestation rates affecting the area assigned to forest land at the beginning of our model run [22].

#### Harvested wood products pool

The carbon stock change on harvested wood products pool up to 2023 was estimated by using the IPCC Tier 2 method [22], using the data reported by FAOSTAT [14] for the following commodities: industrial roundwood (IRW) production, import and export, grouped by coniferous and non-coniferous products (i.e., IRW_C_ and IRW_NC_, respectively); sawn wood (SW) production, import and export, grouped by between coniferous and non-coniferous products (i.e., SW_C_ and SW_NC_, respectively); wood-based panels (WP), paper and paper board (PP) and wood-pulp (WPulp), production, import and export. We first estimated the share of HWP originating from domestic production, distinguished between SW_C_, SW_NC_, WP, and PP. The share of domestic IRW originating from domestic forests is computed considering the production, imports and exports of IRW_C_, IRW_NC_ and WPulp [22]. Then we estimated the annual C inflow to the HWP pool applying to the domestically produced commodities the 2013 IPCC KP Supplement default conversion factors. The C outflow from HWP pool was estimated by applying a first order decay function with constant annual default decay factors for each commodity [22]. A detailed description of this approach is also reported in [46].

From 2024 onward, we applied the same approach described above, but to infer the expected evolution of each commodity within the period 2024 – 2070, we first assessed possible statistical correlations between the production of IRW over time based on the amount of harvest assumed for each scenario, and each commodity. To this purpose, each input data was parametrized based on the following criteria:

- Total annual removals provided by coniferous and non-coniferous species were directly inferred from the CBM model output and based on the total harvest associated to each scenario.
- The share of IRW_C_ and IRW_NC_ products are held constant from 2024 to 2070, based on FAOSTAT data for coniferous and non-coniferous species from 2010–2020 (excluding 2021–2023 due to constant reporting). These shares are 57% of total roundwood for coniferous species and 8% for non-coniferous species. IRW production follows the removals reported by CBM for each scenario from 2024 onwards.
- FAOSTAT data from 2010–2020 for SW_C_, SW_NC_, WP, PP, and WPulp were compared with IRW_C_ and IRW_NC_ (Table 5). When production of a commodity is statistically correlated (r > 0.4) with IRW_C_ or IRW_NC_—such as WP with IRW_C_, and SW_NC_ and WPulp with IRW_NC_ -expected production from 2024 onward will change in proportion to IRW_C_ or IRW_NC_. No such correlation was found for SW_C_ and PP, likely due to higher imports of semi-finished products, so future production for SW_C_ and PP is assumed constant at the 2010–2020 FAOSTAT average.
- Other ancillary parameters such as the share of domestic production attributed to each commodity were assumed to be constant and equal to the latest value reported from FAOSTAT.

## Results

### Historical period 2005—2023

**Fig. 3:**
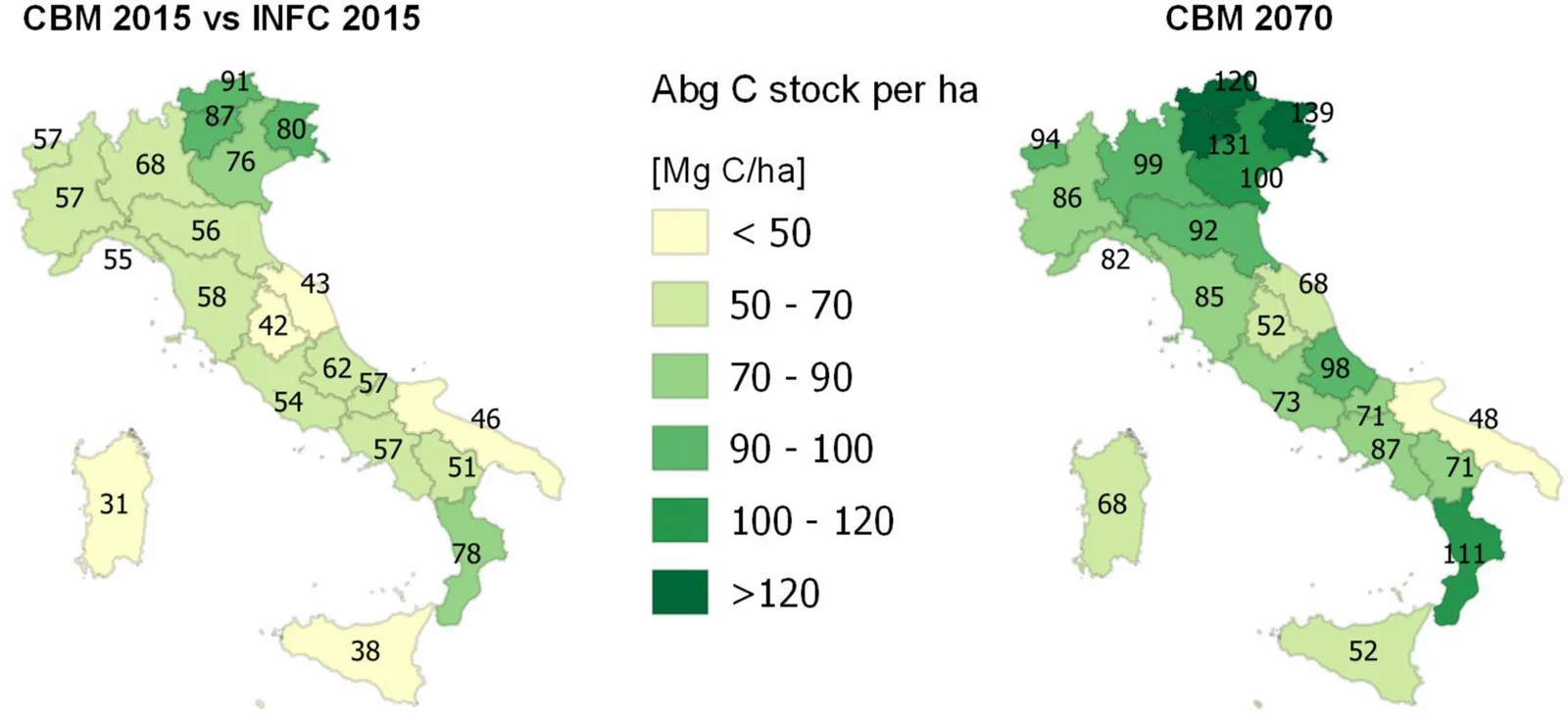
Above ground C stock per ha and region estimated by CBM for 2015 and 2070. Labels on the left panel report the corresponding values reported by INFC 2015; labels on the right panel report the average C stock per ha estimated by CBM for 2070, assuming a fellings rate equal to 0.50

Several parameters—such as merchantable volume, net annual increment, biomass C stock— can be inferred from the model outputs over the historical period 2005–2023. The results calibrated against the average harvest rate (*AvgHistHrv*) can be compared with data reported in the literature, scaled at national (Table 6), regional (Fig. 3) and forest type level (see Supplementary Material, Appendix F).

**Table 6:** Comparison between (i) living biomass volume and C stock, (ii) dead wood volume and C stock, (iii) litter and (iv) soil C stock reported by INFC 2005 and 2015 and the corresponding values estimated by CBM applying the average harvest rate (AvgHistHrv)

Pool	CBM 2005	INFC 2005	CBM 2015	INFC 2015
**Living Biomass volume**				
Merchantable volume (m^3^ ha^−1^)	144.4	144.9 ± 1.5	162.3	165.4 ± 1.7
Net Merchantable Annual Increment (m^3^ ha^−1^ yr^−1^)	3.17	3.19 ^i^	3.19	3.22 ^i^
**Living Biomass C stock** (Mg C ha^−1^)				
Aboveground C stock	56.1	NA	62.1	59.4 ± 0.5
Belowground C stock	13.5		14.3	
Total biomass C stock	69.6		76.4	
**Dead wood volume** (vol. m^3^ ha^−1^)				
Standing dead trees volume	5.2	5.3 ± 0.2	4.9	7.4 ± 0.2
Lying deadwood volume	16.9^ii^	1.9 ± 0.1^iii^	17.9^ii^	5.7 ± 0.2^iii^
Stumps volume	3.3 ^iv^	1.5 ± 0.1	4.1 ^iv^	1.5 ± 0.0
Total dead wood volume	25.4	8.7 ± 0.2	26.6	14.7 ± 0.3
**Dead wood C stock** (Mg C ha^−1^)				
Standing dead trees C stock	1.3	NA	1.3	1.9 ± 0.1
Lying deadwood C stock	3.7^ii^	NA	3.8^ii^	1.1 ± 0.0^iii^
Stumps C stock	0.9	NA	1.1	0.3 ± 0.0
Dead coarse roots C stock	13.8	NA	14.7	NA
Total dead wood C stock	19.7	NA	20.9	3.3 ± 0.1
**Litter C stock** (Mg C ha^−1^)				
L horizon (foliar litter plus dead fine roots < 5mm)	11.4		12.0	
Horizons F, H, O	2.5	3.2 ± 0.0^v^	2.7	NA
Fine and small woody debris plus dead coarse roots (5 ÷ 75mm)	3.6		3.7	NA
Total litter C stock	17.6	3.2 ± 0.0	18.5	N.A
**Mineral soil C stock** (Mg C ha^−1^)	88.1	81.7	88.8	NA

^i^ Data reported by Gschwantner et al. [28] and Avitabile [30], inferred from INFC 2005 and INFC 2015 [28, 30]

^ii^ CBM Medium DOM pool (Coarse Woody Debris)

^iii^ Lying deadwood with diameter and length ≥ 9.5 cm

^iv^ CBM snag branches, including stumps, snag branches and small dead trees

^v^ INFC 2015: horizons L and F, excluding small branches

The carbon pool and the corresponding volume modelled by CBM for several dead wood, litter and mineral soil pools (see Supplementary Material, Appendix F), can be compared with similar data reported by INFC 2005 and 2015 (Table 6).

**Fig. 4:**
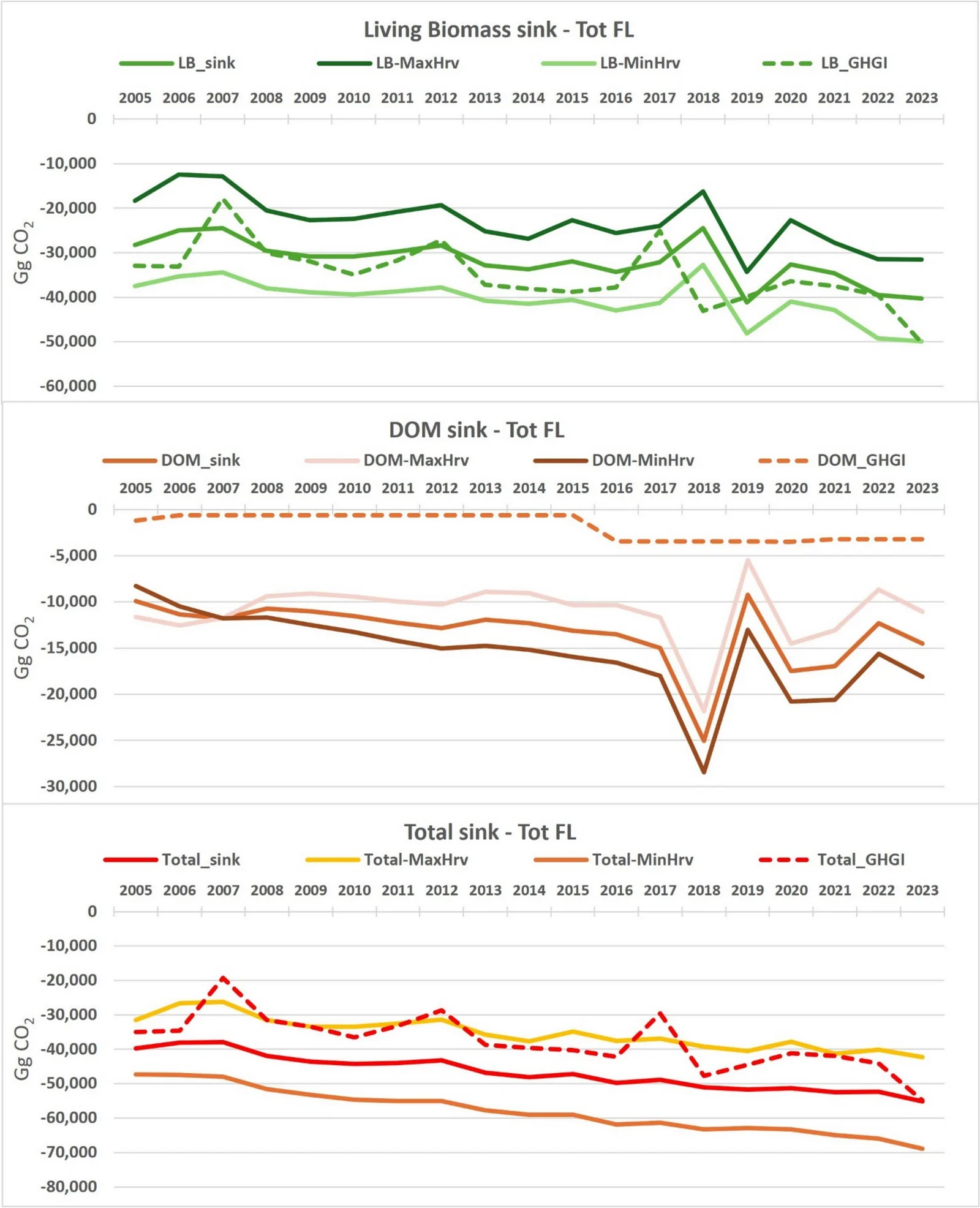
Net forest sink estimated by CBM for living biomass (LB_sink), dead organic matter (DOM_sink, dead wood plus litter) and total (LB_sink + DOM_sink + soil organic matter) C pools, compared against the C sink reported by the Italian GHGI 2025 [44]. Minimum (Min) and Maximum (Max) LB, DOM and Total values refer to MinHistHrv and MaxHistHrv harvest levels, respectively (see Table 1). All data refers to Total Forest Land. All the values are reported from an atmospheric perspective, i.e., negative values represent a sink and positive a source

Figure 4 reports the net forest C sink estimated by CBM for living biomass, dead organic matter and total C pools, within the historical period 2005 – 2023, further compared against the total, DOM and biomass C sink reported by the Italian GHGI [2].

**Fig. 5:**
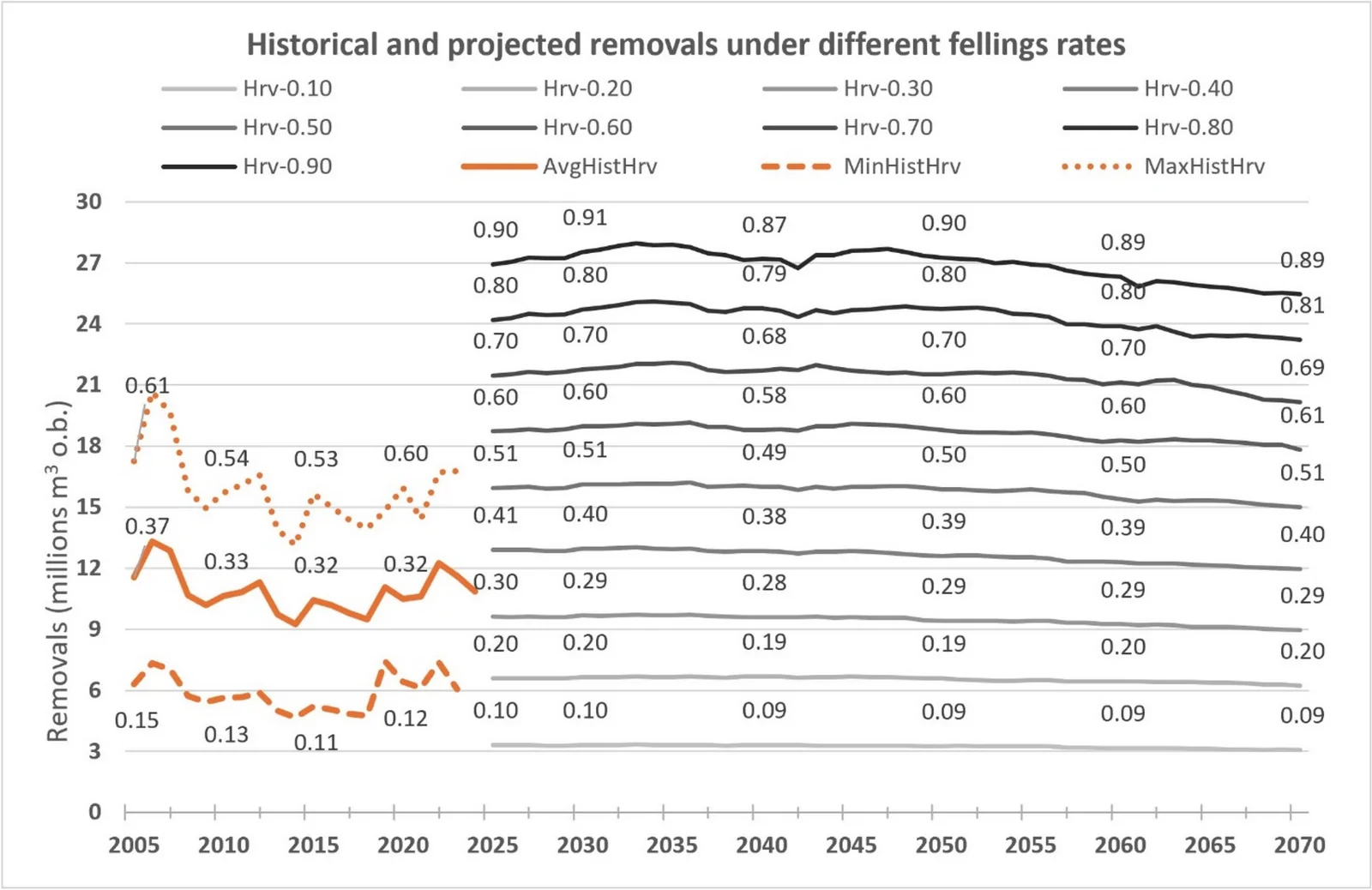
Historical, until 2023, and projected (from 2025 to 2070) evolution of total removals under different harvest scenarios, corresponding to an average (AvgHistHrv), minimum (MinHistHrv) and maximum (MaxHistHrv) harvest level applied to the historical period 2005 – 2023, as inferred from [7], and to an increasing fellings rate from 0.10 to 0.90 of NAI determined on the forest area available for wood supply. Labels report the evolution of the fellings rate as estimated by model under different harvest levels

By varying the average amount of harvest applied to the historical period according to Fig. 5, the living biomass C sink varies, on average and excluding HWP, by about ± 28%, when compared to the intermediate harvest level (Fig. 4). The average total forest C sink estimated by model changes from about -48 Tg CO_2_ yr^−1^, assuming the intermedium harvest level, to -57 and -35 Tg CO_2_ yr^−1^, when applying the lowest and highest harvest rate, respectively.

### Projection period 2024—2070

**Fig. 6:**
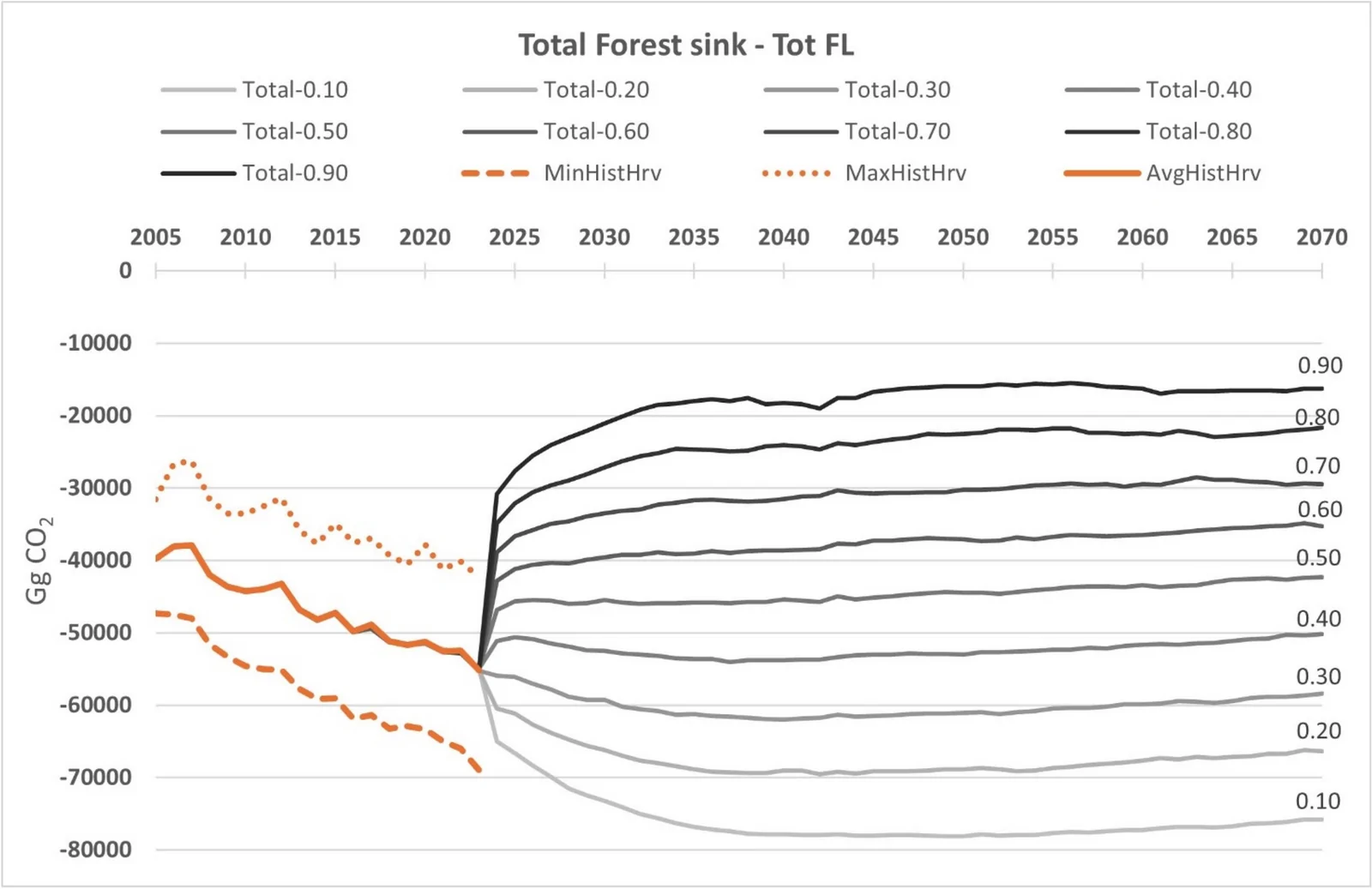
Total forest C sink estimated by model within the entire simulation period 2005 – 2070, according to different harvest levels, within the historical period 2005–2070, and felling rates (also reported by labels), within the period 2024 – 2070. All the values are reported from an atmospheric perspective, i.e., negative values represent a sink and positive a source

By varying felling rates between 0.10 to 0.90 of the NAI for FAWS, we estimated that, in 2070, the total forest C sink varies between -76 Tg CO_2_ yr^−1^ and -16 Tg CO_2_ yr^−1^ (Fig. 6). If the projected harvest rate is maintained at the same average level estimated in 2023 (i.e., around 0.30 and 0.40 corresponding to *AvgHistHrv*), the total C sink would range between -50 Tg CO_2_ yr^−1^ and -58 Tg CO_2_ yr^−1^, in 2070.

**Fig. 7:**
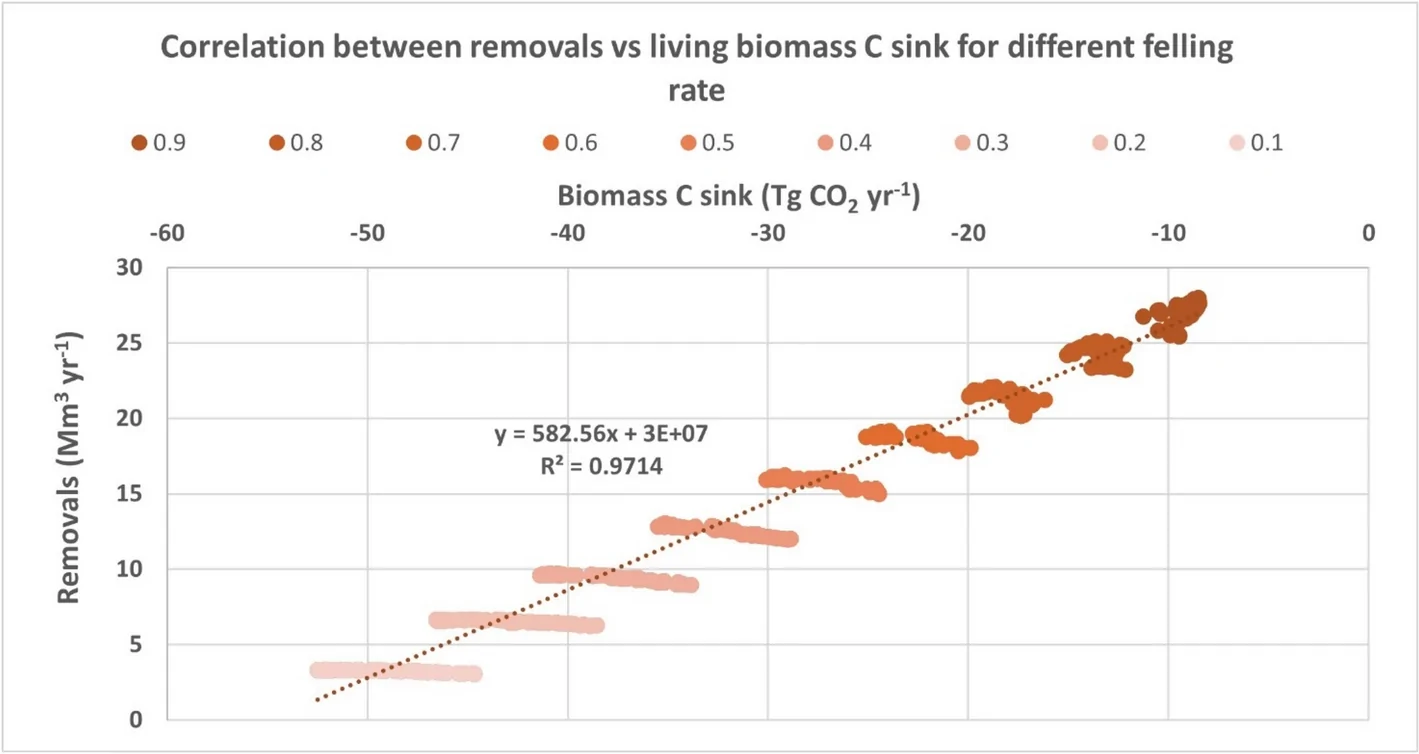
Correlation between total removals, as determined under different felling rates scenarios and modelled living biomass C sink within the period 2024 – 2070

As expected, the living biomass C sink over the entire simulation period is clearly dependent on the absolute amount of harvest associated with different fellings rates (Fig. 7).

### Harvested wood products pool

**Fig. 8:**
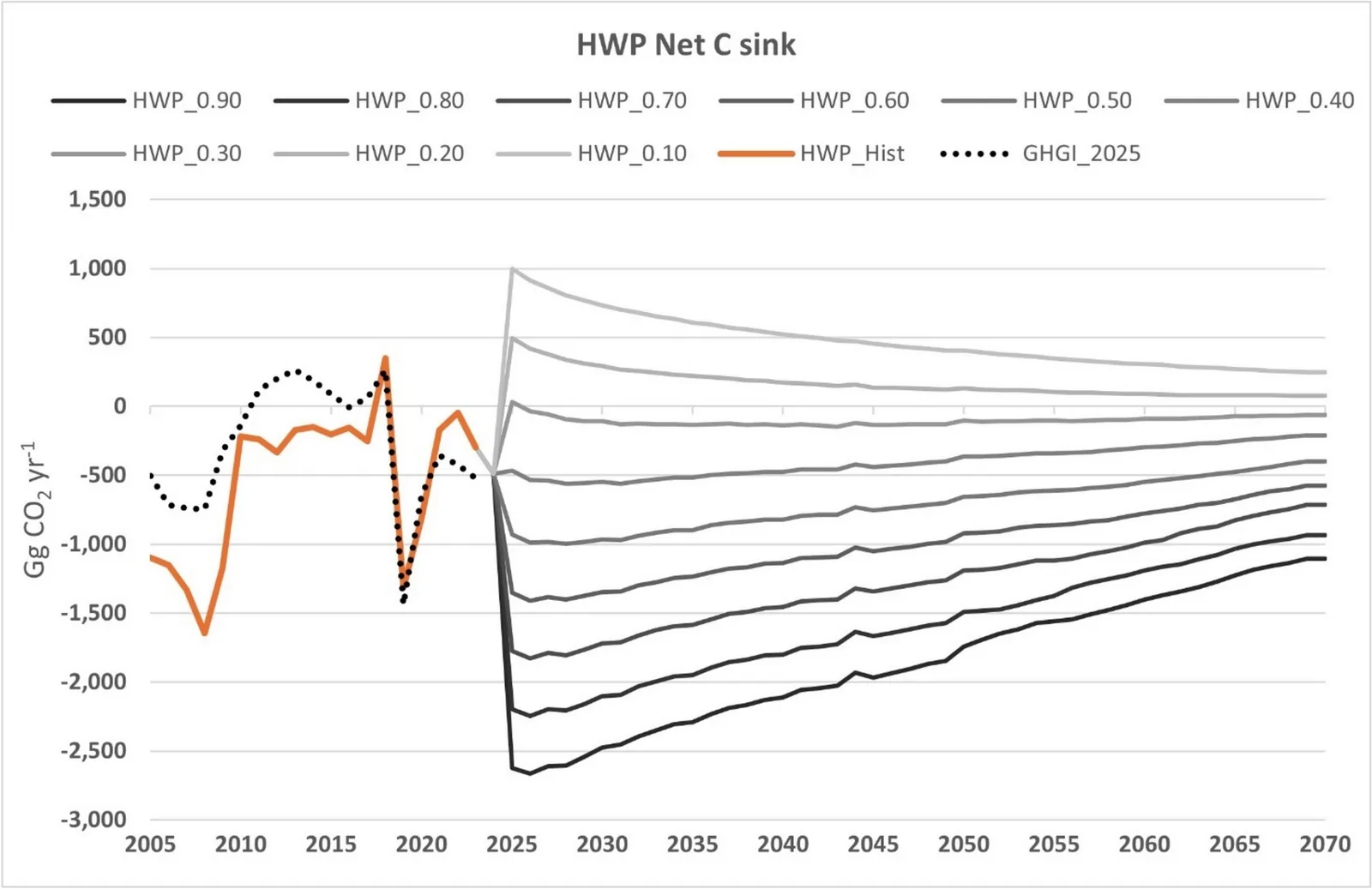
Harvested Wood Products net C sink estimated within the historical period 2005 – 2023, based on the input data reported by FAOSTAT 2025 [14] and under different harvest scenarios within the period 2024–2070. The plot highlights net removals as reported for the HWP pool by the Italian GHGI (2025). All the values are reported from an atmospheric perspective, i.e., negative values represent a sink and positive a source

Based on the data reported by FAOSTAT [14], over the period 2005 – 2023 about 77% of removals were provided by broadleaves species, with the remaining part provided by coniferous species. At the same time, only 23% of total removals, provided both by broadleaves and coniferous species, were used as industrial roundwood. Because no other alternative data sources are available for Italy, the mitigation potential attributed to HWP pool can only be based on these data. The resulting HWP C sink, reported in Fig. 8, is equal, on average to -548 Gg CO_2_ yr^−1^ over the historical period.

From 2024 onward, we aligned our analysis assumptions with the data reported by FAOSTAT, by assuming that about 74% of removals are provided by broadleaves species. Based on this assumption, the HWP mitigation potential varies between 2024 and 2070 proportionally to the absolute amount of harvest applied by model.

## Discussion

### Historical period 2005—2023

#### Volume, biomass and carbon stock

At the national level, the CBM model shows a good agreement with merchantable volume reported by INFC data (Table 6). Some differences emerge at regional (see Fig. 3, left panel) and forest types level (see Supplementary Material, Appendix F). For instance, in the case of Beech (FS) and Chestnut (CS), the modelled estimates fell outside the INFC 2015 error range. These local discrepancies, characterized by both over- and underestimations depending on the FT, tend to compensate each other, leading to a well-balanced and reliable estimate at the national scale. The aboveground C stock is slightly higher—by about 4.5% — than the values reported by INFC 2015. These differences suggest there may be some inconsistency between the conversion factors (i.e., biomass allometric equations and wood density values) used by the model and those considered in the INFC data.

A comprehensive analysis of increment trends over time can be conducted based on the model output. During the period 2005–2023, the mean NAI was 2.97 m^3^ ha⁻^1^ yr⁻^1^. Over this timeframe, approximately 30% of NAI was extracted through felling operations (applying the *AvgHistHrv* level), with the remaining portion -equivalent to 2.09 m^3^ ha⁻^1^ yr⁻^1^ -contributing to the net merchantable biomass carbon sink. The derived increment from INFC 2005 and INFC 2015, established using a standard EU reference definition [28, 30], is equal to 3.19 and 3.22 m^3^ ha⁻^1^ yr⁻^1^, which closely aligns with the simulated NAI from CBM (Table 3).

Three of the four dead wood biomass sub-pools considered by CBM are directly comparable with INFC data. For 2015, the modelled standing dead tree volume, derived by wood density coefficients reported in the literature [47, 48] is lower (4.6 m^3^ ha⁻^1^), than values reported by INFC 2015 (7.4 ± 0.2 m^3^ ha⁻^1^), but, when expressed as carbon stock, the difference between the model estimate (1.3 Mg C ha⁻^1^) and INFC 2015 data (1.9 ± 0.1 Mg C ha⁻^1^) is marginal.

The C stock estimated for the medium DOM pool (3.8 Mg C ha⁻^1^) is approximately three times higher than the value reported by INFC 2015 for lying deadwood (1.1 Mg C ha⁻^1^). This discrepancy likely arises from the different biomass components and thresholds adopted by the two systems. INFC data are derived from direct field measurements of lying deadwood with a minimum diameter of 10 cm. In contrast, the CBM Medium DOM pool is parameterized according to Canadian National Forest Inventory standards, where coarse woody debris includes downed dead wood with a diameter > 7.5 cm (see for example, [49]). Since the decay rate of woody debris is exponentially related to its diameter [50], a relatively small difference in the minimum threshold (≈ 2 cm) can significantly affect the pool size. Apart from the volume and corresponding C stock attributed to stumps, and the fact that CBM estimates also include snag branches and small dead trees, the total C stock estimated by CBM is mostly due to dead coarse roots, which is not measured by INFC. Specifically, the modelled C stock attributed to Belowground Fast DOM pool (representing coarse dead roots > 5 mm) reached values of 13.8 Mg C ha^−1^in 2005 and 14.7 Mg C ha^−1^in 2015. This specific component is not considered in INFC field assessments; however, our estimates are consistent with empirical studies on mature forest stands. For instance, Palviainen et al. [51], in a 40-year chronosequence study on *Picea abies*, reported that the combined C stock of stumps and coarse roots (scaled to carbon content) can range between 8.0 and 10.7 Mg C ha^−1^. It is crucial to emphasize the high uncertainty and the methodological challenges inherent in measuring the deadwood pool, as field surveys often do not capture the full extent of underground dead coarse roots [51].

The modelled total C stock for dead wood pool varies between 19.7 and 20.9 Mg C ha^−1^, in 2005 and 2015, respectively. We acknowledge that these values are higher than those typically reported in the literature for Italian forests, which do not measure dead coarse roots. However, we notice that specific studies which provide a more accurate assessment of the dead wood C pool report on a considerably larger C stock ranging between 40 and over 60 Mg C ha^−1^ for temperate old-growth forest sites [52].

While the average carbon stock modelled by CBM for soil (88 Mg C ha⁻^1^ over the entire historical period) is consistent with data measured by INFC and reported by literature [53], the modelled C stock for the litter pool—averaging 18.2 Mg C ha⁻^1^— is considerably higher than empirical observations. For instance, INFC 2005 reports a litter C stock of 3.2 Mg C ha⁻^1^. Similar to the discrepancy observed for deadwood, this divergence is inherently methodological. While the CBM definition of the litter pool is broader, effectively encompassing fine and small woody debris as well as dead coarse roots, the INFC field protocol strictly restricts litter collection to epigeal organic horizons (i.e., OL, OF, and OH layers), thereby omitting the underground structural root fraction [54].

#### Carbon stock change

For the historical period 2005–2023, the modelled living biomass C sink increases from -28 Tg CO_2_ yr^−1^ in 2005, to -40 Tg CO_2_ yr^−1^ in 2023, which is consistent with the trend reported by the Italian GHGI [2]. The increasing biomass sink is mostly due to a decrease in harvest up to 2018 and with a low net afforestation rate up to 2015, followed by a significant increase over the period 2016 and 2023. The modelled aboveground biomass sink can also be compared with the corresponding carbon stock change inferred from INFC 2015, which ranges from -29 to -31 Tg CO₂ yr⁻^1^ (see Supplementary Material, Appendix F3).

Annualization of derived stock change estimates is challenging, however, we can assume that the average C stock change inferred from INFC 2015 can be broadly referred to the period 2015–2017. During this timeframe, the CBM estimate of the aboveground carbon sink ranges from -27 to -29 Tg CO₂ yr⁻^1^, which aligns with the interval derived from INFC data. Notably, the CBM incorporates direct CO₂ emissions resulting from wildfires occurring within the same timeframe—factors that cannot be directly accounted by INFC measurements. By assuming that all living biomass stock in areas affected by wildfires have been completely lost (i.e. oxidized with direct GHG emissions to the atmosphere), the Italian GHGI reports strong interannual variations on the living biomass pool within the years affected by major fires events (i.e. 2007, 2012 and 2017). In contrast, CBM does not report similar interannual variations on living biomass pool, because the area affected by wildfires has been preliminary distributed between surface fires, mostly affecting DOM pools and only 20% of the merchantable living biomass, and crown fires, affecting 80% of the living biomass pool. In this way, the model assumptions reduce the impact of large wildfires on the forest C sink, when compared to GHGI data.

The 2018 windstorm, which affected 9.5 million m^3^of the standing volume, resulted in an abrupt transfer of about 2 mil Mg C from living biomass to DOM pool and a correspondingly low living biomass sink estimates in the same year ( -25 Tg CO2 yr-1). These findings are consistent with similar studies on the effect of major windstorms on the forest biomass carbon sink, such as those observed in France in 1999 and, more recently, in Czechia since 2018 [55, 56]. In 2018, the Italian GHGI [2] shows a contrasting response, with the biomass C sink increasing from a minimum value of -25 Tg CO_2_ yr^−1^ in 2017 (mostly due to the occurrence of wildfires in the same year) to -43 Tg CO_2_ yr^−1^ in 2018, without any apparent effect on living biomass or DOM pool due to the windstorm, which is not accounted within the Italian GHGI. In subsequent years, the modelled C sink is driven by the combined effects of interannual variation in harvests, mostly due salvage logging, and further losses from living biomass due to insect outbreaks. The effects of these disturbance events were modelled in detail for our analysis, including the definition of input data, at NUTS 2 level. The model considers most recent information from the literature and data collected at regional level (see Appendix E). Despite the described different methodological assumptions, the overall biomass C sink from 2020 onward is well aligned, when the modelled and Italian GHGI data is compared.

The modelled DOM C sink increased steadily from about -9 Tg CO_2_ yr^−1^ in 2005 to -13 Tg CO_2_ yr^−1^ in 2017. This pattern is mostly driven by the turnover rate associated with the increasing stock available on living biomass (+ 11% between 2005 and 2015). This model-based trend aligns well with national forest inventory data. Indeed, despite its inherent uncertainty, the total deadwood volume measured by INFC increased by approximately 67% between INFC 2005 and INFC 2015, which is consistent with the model results (Table 6). In 2018 there is an abrupt shift in the allocation of living biomass to dead wood and litter pools due to the effect of the windstorm, which further increases the DOM C sink. From 2019 onwards, part of the biomass stock is transferred from dead wood to products pools due to salvage logging, which reduces the overall DOM C sink. Over the same time period, however, the modelled living biomass sink increases because of the reduced pressure of ordinary silvicultural treatments on the three regions mostly affected by the windstorm and by the following bark beetle attacks. These impacts are not reported in the Italian GHGI, where the DOM C stock is derived from constant, linear accumulation rate, based on INFC field measurements [2]. This may also explain the considerably lower DOM C sink reported by Italy (on average -1.7 Tg CO_2_ yr^−1^), when compared with the values estimated by our model (about – 11 Tg CO_2_ yr^−1^ over the entire historical period). This marked discrepancy stems from the GHGI’s static approach based on linear accumulation rates and more restrictive deadwood definitions, whereas the CBM dynamically accounts for natural disturbance-driven transfers and belowground fractions. Consequently, current GHGI sink estimates are highly conservative, highlighting the importance of integrating more dynamic modeling frameworks into inventory reporting.

The C stock changes in mineral soil is assumed to be zero by Italian GHGI, at least for the category FL-FL, while for L-FL Italy applies the IPCC default method [2]. In case of CBM, within the historical period mineral soil is expected to be a stable pool, with a net C sink equal to about -1 Tg CO_2_ yr^−1^.

The modelled total C sink increases from -40 Tg CO_2_ yr^−1^ in 2005 to -55 Tg CO_2_ yr^−1^ in 2023, driven by the increasing sink of living biomass and DOM pools, which provide, on average, about 68% and 25% of the total C sink, respectively. The share of the C sink between biomass and DOM pools is consistent with the values reported by other countries (i.e., Czech Republic) applying similar approaches [56]. The lower C sink reported by the Italian GHGI is mostly due to DOM pool, that provides, on average, only 5% of the total C sink reported by Italy over the same period. Further differences are due to interannual variations affecting the living biomass pool, mostly due to wildfires.

In general, we can conclude that the living biomass sink simulated by CBM, is mostly driven by (i) harvest demand, (ii) natural disturbances and consequent salvage logging, above all after the 2018 windstorm, and (iii) the increasing contribution of afforested lands. Since 2016 onward, according to the latest Italian GHGI, the annual afforestation rate has abruptly increased from about 40 kha yr^−1^ to over 180 kha yr^−1^. Over the period 2005 – 2015, the annual rates of afforestation and deforestation used in our study were determined by directly comparing, for each forest type and region, the area reported by INFC 2005 and INFC 2015. The resulting afforestation rates differ from the one reported by the Italian GHGI, where the annual afforestation and deforestation rates are equal, within the same period, to about 49 kha yr^−1^ and 4 kha yr^−1^, respectively [2]. Between 2016 and 2023, the annual afforestation rate has considerably increased, because our modelling exercise aligns with the forest area reported by the Italian GHGI. We notice that, in 2023, the C sink related to the cumulated afforested land area (i.e. 1,954 kha between 2005 and 2023) is about -13 Tg CO_2_ yr^−1^ (this corresponds to about 30% of the total C sink estimated by our model). The latest Italian GHGI, which reports about 2,315 kha for L-FL in 2023, reports -14 Tg CO_2_ yr^−1^, which is well aligned with our estimates, and correspond to about 25% of the C sink attributed to total FL [2].

If one assumes an average harvest rate of 11 Mm^3^ yr^−1^, our estimates for the living biomass pool, and the corresponding C sink, are quite well aligned with the data reported by Italy. All of these results, however, are inherently affected by the uncertainty on the total amount of harvest occurring in Italy over the period 2005—2023. Indeed, according to the latest literature [7], the uncertainty attributed to total fellings ranges by ± 50%. As suggested by other studies [9, 11], harvest statistics are generally underestimated in Italy, therefore we can reasonably assume that the forest C sink ranges between the estimates corresponding to the average and the upper (*MaxHistHrv*) harvest levels assumed in our analysis.

By comparing the average, upper and lower amount of estimated felling rates with NAI inferred from the model output for the area available for wood supply, we estimate that the average felling rate varies from 0.33, assuming the intermediate harvest level, to 0.12 and 0.56, assuming the lower and upper harvest rate, respectively (Fig. 5). With most of the other European countries reporting a felling rate higher than 0.60 for 2010 and 2015 [18], we notice that the latest value estimated in our study (0.56) is the only one aligned with the statistics reported by other countries conterminous with Italy.

For HWP C pool, our modelled time series captures the same inter-annual dynamics as the official Italian inventory; however, the absolute values differ, with our estimates indicating a mean HWP C sink 94% higher than the average reported in the latest Italian GHGI [44] (-283 Gg CO₂ yr⁻^1^). Nevertheless, both the assessments confirm that the relative contribution of the HWP (representing less than 2% of the living biomass C sink) is considerably lower than the values reported by other European countries, where HWP pool generally provides about 10% of the C sink additional to living biomass [46]. The lower HWP share in Italy is likely due to the relatively low amount of domestic wood used for industrial roundwood production, as inferred from official statistics reported by FAOSTAT.

Within our modelling framework the distribution of harvest between different administrative regions is inferred by [7] and the share of harvest provided by broadleaves species varies, over the historical period, between 61 and 50%, being generally lower after 2018 due to salvage logging, mostly affecting coniferous species. Even if this share is partially different from FAOSTAT statistics, we could not harmonize the estimates provided on the HWP pool with our model output, because historical data on semi-finished commodities, which are essential for applying the IPCC [22] approach, can only be inferred from FAOSTAT database.

### Projection period 2024 – 2070

For the simulation period from 2024 to 2070, the forest C stock continuously increases, under all model scenarios, both at national and regional level (see the example reported in Fig. 3). The C sink at the end of the projection period varies between -76 Tg CO_2_ yr^−1^ and -16 Tg CO_2_ yr^−1^, under the lowest (0.10) and highest (0.90) felling rates, respectively (Fig. 6). Since the absolute harvest volume was determined — based on our assumptions — by the harvest rate (i.e., the ratio of fellings to NAI) and given that NAI was derived from the model output, the C sink estimates across different harvest rate scenarios appear remarkably stable. This stability arises because the absolute harvest amount adjusts dynamically throughout the projection period, according to the evolution of NAI, which is, in turn, also determined by the age-structure (Fig. 5). Specifically, each scenario was implemented through an iterative approach to maintain a consistent harvest rate; consequently, within the 2024–2070 projection period, the harvest rate exhibits some minor variations (± 0.02) across all scenarios.

Since we excluded further land-use change, windstorms and bark beetle outbreaks, the living biomass C sink, over the projection period, is mostly dependent on the absolute amount of harvest associated with different felling rates (Fig. 7). We notice that when the maximum harvest rate is considered over the projected period (e.g. over 25 Mm^3^ yr^−1^), the biomass C sink converges at a minimum sink level, i.e. about -10 Tg CO_2_ yr^−1^ for the entire simulation period (Fig. 7). At the same time, our model results suggest that lower harvest rates may condition future C sink dynamics, potentially leading to larger variations in response to shifting forest age structure. This means that, if lower harvest ensures a higher C sink over long time periods, they can also cause larger variations (i.e. reductions) of the C sink. In this case, the natural aging process is not counterbalanced, at least for FAWS areas, by forest management activities (i.e. harvest), which can contribute to rejuvenating the age structure. This is true both for the even-aged and the uneven-aged forest structures, and even more, for the large forest area covered, in Italy, by aging coppices [6]. Other factors, however, can further complicate this dynamic. Pure even-aged high forest stands can be more prone to insects outbreaks, windstorm damage and ageing processes, which may abruptly reduce the forest living biomass C pool. In contrast, young even-aged stands derived from clear-cuts, mostly from coppices in case of Italy, may have negative impacts on soil and DOM pools.

The inflow of wood material to HWP pool is less than the outflow when the harvest rate is below 0.30, and the HWP pool becomes a source of C to the atmosphere (at least if we assume steady-state conditions for the share of domestic production attributed to each commodity). As expected, the highest harvest rates also provide the highest HWP mitigation potential. This can partially compensate for the lower C sink assigned to living biomass pool. Assuming the highest felling rate (0.90 of NAI) is applied, the C sink due to the HWP pool increases, on average, by 20% the C sink attributed to living biomass pool.

### Strengths and weaknesses

#### Model calibration

We successfully validated our results using various data sources, partially independent from input data used for model calibration (mostly provided by INFC 2005 [6]). Our results reproduced, for the living biomass pool, the same multi-annual pattern as the Italian GHGI data [2], but we report a quite different dynamic, when the impact of natural disturbances is considered. Indeed, our study suggests that wildfires, and, more importantly, windstorms and bark beetle outbreaks result in a reallocation of C from living biomass to dead wood and litter pools, which increases the net C sink associated to DOM pool. On the contrary, by assuming a linear accumulation rate of DOM C stock without any additional transfer due to external disturbance events, the Italian GHGI attributes a constant C sink to DOM pool. This explains the final difference between the total C sink estimated by our study, -47 Tg CO_2_ yr^−1^ over the historical period 2005 – 2023, and the data reported by the Italian GHGI (-38 Tg CO_2_ yr^−1^).

To our knowledge, this is the first study that quantifies the inherent uncertainty due to the assessment of historical and current harvest rate on the forest C sink (Fig. 4). Accounting for a ± 50% harvest uncertainty derived by literature, the total C sink at the country level ranges from -58 Tg CO_2_ yr^−1^ and -35 Tg CO_2_ yr^−1^. Given an average historical sink of -47 Tg CO_2_ yr^−1^, this uncertainty in harvest rates translates into a ± 24% variation in the total C sink This means that, depending on historical harvest rates, the average total C sink may vary, at country level, by -16% ÷ + 18%. Crucially, the lowest net C sink (i.e., -35 Tg CO_2_ yr^−1^) represents the most realistic estimation, given the well-documented underreporting in official Italian harvest statistics [9, 11, 57].

The overall uncertainty of this evaluation, however, is also amplified by our assumptions regarding the relative amount of felling residues, which were preliminarily set at a constant, general level equal to 15% of total harvest. Undoubtedly, a more accurate assessment, scaled at the species and regional level, would refine our analysis.

The CBM modelling framework facilitates the annual transfer of C from living biomass to seven DOM sub pools, based on a series of predetermined annual turnover rates. Despite our effort to partially revise DOM parameters, using ancillary information reported by literature, we are aware that further assessments, possibly based on direct field measurements collected at national and local level, are needed to improve our model capacity, and further validate our results. For example, although temperature generally overrides species diversity in controlling wood decomposition [58], our climate-decay relationships were restricted to broadleaved species. Expanding these assessments to coniferous species will therefore be a key step to reduce modeling uncertainty.

The utilization of two distinct datasets for growth curve derivation is justified by the dual role of these curves in model initialization and simulation. Growth curves for model initialization were primarily derived from static standing volume data, whereas increment data were employed to calibrate the growth dynamics during the simulation phase. In both instances, we prioritized data stratified by forest type, management type and region. However, due to the limited availability of age-stratified data reported by INFC, some analyses had to be upscaled to the national level. Consequently, for certain FT and MT, regional-specific growth curves could not be defined. Furthermore, the interpolation of the original volume and increment data —achieved through combined exponential and power functions — may not fully capture the variability across all age classes. Although we implemented rigorous data cleaning protocols to ensure consistency, excluding outliers and poorly fitting curves, the sheer complexity and the large number of derived growth curves mean that minor inconsistencies cannot be entirely ruled out. These uncertainties in growth curve derivation represent a structural component of our model and should be considered when interpreting the simulated carbon dynamics, above all if scaled at FT or local (i.e. regional) level.

With this study we also improved our model capacity on uneven-aged forest stands. These forests cover about one third of the total forest area in Italy, and they were modelled by developing ad-hoc growth curves, derived by simulating the evolution of current annual increment against volume, as reported by INFC 2015. Accordingly, these new growth curves explicitly represent the anticipated temporal progression of increment relative to volume, as determined by the actual management practices implemented in Italy over recent years (directly assessed by INFC field measurements carried out between 2016 and 2019). Each uneven-aged stand, categorized by forest type and region, is assigned to a volume class based on the average volume and increment as reported by INFC 2005. The stand follows the projected changes in these variables throughout the model run, while partial cuts, eventually applied during the model run, reduce aboveground biomass by 15%-20% without altering the general progression along the growth curve.

#### Future model scenarios

The detailed assessment of our results for the historical period 2005—2023 preceded the sensitivity analysis performed on the following period 2024 – 2070. This approach was aimed at quantifying the impact of various harvest levels on the long-term evolution of the forest C sink. In this case, rather than considering different possible economic scenarios, which are unavoidably uncertain, we tested the impact of various felling rates, directly determined by our model, by considering the ratio between fellings and NAI on the forest area available for wood supply. This evaluation ensured, on one side, a conservative management approach on FNAWS, excluding protected forest areas from any silvicultural intervention, and on afforested lands, which won’t be harvested before 2070, because, under the continuation of current silvicultural practices, they do not reach the minimum age for final cut. On the other side, we prevented a possible overexploitation of forest resources on FAWS by varying each future harvest scenario according to the NAI as determined on the forest area available for wood supply. This means that, when the NAI is decreasing over time, because of the ongoing aging process or disturbances, the absolute amount of harvest will decrease accordingly.

It is worth noting that our projections do not account for future natural disturbances (e.g., windthrows and insect infestations). Incorporating these stochastic events into a long-term deterministic model introduces high parameter uncertainty. However, given the well-documented trend of increasing climate-driven disturbances, this assumption likely leads to an overestimation of the forest carbon sequestration capacity. The quantified sink under the proposed scenarios should therefore be interpreted as a theoretical potential, which could be significantly downscaled by future extreme events.

#### Harvested wood products

The additional analysis performed on the HWP pool, confirmed that the relative contribution of harvested wood products pool to the total C sink is negligible within the historical period 2005 – 2023, due to the specific conditions of the Italian forest supply chain, with most of the harvest used for energy [14]. For the projected period, where felling rates are ≤ 0.30, the HWP pool slightly decreases the total C sink. With the highest felling rate, the total C sink increases to -17 Tg CO_2_ yr^−1^ in 2070, when adding the HWP pool. Our projections of harvested wood products beyond 2024 are based on statistical correlations between historical commodity production and roundwood production. However, the use of relatively low correlation thresholds (r > 0.4) and the assumption of proportional relationships (or constant shares of certain commodities over a long-time horizon) may not adequately capture the complex drivers of HWP markets. Indeed, by assuming a steady-state condition of the forest supply chain (e.g., a constant fraction of IRW assigned to domestic production and to fuelwood), we excluded the impact that structural changes in markets, technology and trade patterns may have on the HWP pool. Recent studies suggest that the HWP carbon stock can be increased in the short-medium term by prioritizing the use of wood for material purposes, while maintaining constant harvest [59]. Nevertheless, we stress that this would require in-depth structural changes to the forest supply chain, such as those dependent on external market conditions, which could only be achieved over an extended period of time. We are also aware that harvest statistics data reported by FAOSTAT as used for the present study are largely uncertain and probably underestimated [7].

Material and energy substitution benefits arising from the use of wood products to replace carbon-intensive alternatives were excluded from this study. However, it is reasonable to assume that these benefits will decrease over time as competing industrial sectors undergo progressive decarbonization [60].

#### Climate change

Although our long-term simulations already included the impact of wildfires, we did not consider the potential further impacts of additional disturbance events—such as those that occurred between 2018 and 2022—and exceptional fire events. We are aware that, due to the increasing impact of climate change, the frequency and magnitude of these events are expected to increase in the future. In this case, since the impact of extreme events, such as windstorms or bark beetle outbreaks on coniferous stands, is often age-dependent, the model-driven trend toward older forest structures under reduced harvest rates suggests a potential increase in the theoretical susceptibility of these ecosystems to such events. At the same time, we did not consider the expected impact that climatic drivers (e.g., droughts and increasing mean annual temperature) may have on the short to medium term evolution of the forest C sink. This has already been assessed by other studies [27] and it has been reported by other European countries [61, 62] as one of the main drivers affecting the recent evolution of the forest C sink. Climate change is also expected to modify species distribution and composition, promoting a partial substitution of even-aged coniferous stands, with broadleaves and mixed stands [63]. Our modelling assessment partially accounted for this process, by assuming that after major disturbance events affecting some alpine region in 2018, spruce and fir even-aged stands are partially replaced by predominantly beech stands. This is a possible adaptative forest management strategy, to address climate change impacts [64]. This process, involving species substitution, however, was not further expanded, from 2024 onward, because we assume that it can be mostly linked to cyclical disturbance events, with long term return intervals (e.g., windstorms and bark beetle outbreaks), not considered within our timeline. Due to the specific characteristics of Italian management system, mostly oriented towards thinnings and small-scale intervention systems for coniferous stands, promoting active, large-scale species-substitution interventions, such as expected within other European countries [56], is quite challenging and it would probably require specific legislative initiatives.

## Conclusions

This study introduces a novel bottom-up modelling framework designed to refine the estimation of Italy’s forest carbon sink by addressing historical inconsistencies in harvest statistics. By integrating NUTS2 data from the INFC 2005 with updated growth curve parameterizations— also derived from INFC 2015 plot-level data—and DOM parameters calibrated against literature-based values, our approach overcomes the limitations of previous top-down assessments, providing a more granular and reliable representation of the Italian forest carbon balance.

Our results highlight the high sensitivity of the carbon sink to the interplay between biomass accumulation (in both living and DOM pools), harvesting intensity, and —during the historical period — natural disturbances. When validated against other data sources – only partially independent in case of INFC 2005—the model demonstrates a strong capacity to capture inter-annual dynamics. However, the observed discrepancies in the litter and dead wood pools — where model estimates exceed inventory snapshots — underscore the need to better account for legacy carbon dynamics that are typically under-sampled in standard field protocols.

Despite these advancements, this research acknowledges two critical constraints. First, our long-term harvest scenarios assess sustainability primarily through a management lens, based on wood supply availability; these should be viewed as technical benchmarks rather than exhaustive ecological assessments, as they currently omit the complex feedback loops associated with climate change and natural disturbance regimes [64]. Second, while our bottom-up framework is potentially transferable to other European countries, its broader applicability depends on the availability of harmonized inventory data and the ability to adapt soil and deadwood turnover parameters to local environmental conditions.

Ultimately, this study provides an informed modelling framework to support the discussion surrounding the new Italian forest strategy. By quantifying the uncertainty linked to harvest statistics, we offer a strategic outlook that can assist policymakers in aligning forest management with long-term climate mitigation goals. Future research should prioritize the integration of climate-sensitive growth models and stochastic disturbance modules to transition from management-based benchmarks toward a comprehensive assessment of forest resilience and carbon sequestration potential in a changing climate.

## Supplementary Information

Additional file 1.

Additional file 2.

## Data Availability

Input data used for this study is mostly collected by publicly available databases. The datasets used and/or analyzed during the current study are available from the corresponding author on a reasonable request.

## Abbreviations

AIDB: Archive index database
AR: Afforestation
C: Carbon
CBM: Carbon Budget Model
CLU: Climatic Unit
CRT: Common Reporting Tables
D: Deforestation
Dbh: Diameter at breast height
DOM: Dead organic matter
EU: European Union
FAWS: Forest area available for wood supply
FISE: Forest Information System for Europe
FL-FL: Forest land remaining forest land
FNAWS: Forest area not available for wood supply
FT: Forest type
GHGI: Greenhouse gas inventory
HWP: Harvested wood products
INFC: National forest and carbon inventory
IPCC: Intergovernmental panel on climate change
IRW_C_: Industrial roundwood conifers
IRW_NC_: Industrial roundwood non conifers
L-FL: Land converted to forest land
LULUCF: Land use, land use change and forestry
NAI: Net annual increment
NUTS: Nomenclature of territorial units for statistics
PP: Paper and paper board
SW_C_: Sawn wood conifers
SW_NC_: Sawn wood non-conifers
WP: Wood panels
WPulp: Wood-pulp
