# Supplementary material for: The Italian forest carbon sink: historical evolution and future projections under alternative harvest scenarios
Source: Carbon Balance Manag. 2026 Jul 18;21:127. doi: 10.1186/s13021-026-00486-6 (PMC13670799; doi:10.1186/s13021-026-00486-6)
Supplement: Supplementary file 1 [file 13021_2026_486_MOESM2_ESM.docx]

## Supplementary Materials

### Appendix A: general model assumptions

Figure S 1: tThe following flowchart summarizes the main steps and methodological improvements applied within the present study for the preliminary model calibration

Table S 1: Italian administrative regions/autonomous provinces, with the corresponding NUTS code and acronym, as used by CBM model within the present study

| **Regions/provinces** | **NUTS 2 code** | **CBM acronym** |
| --- | --- | --- |
| Abruzzo | ITF1 | **Ab** |
| Basilicata | ITF5 | **Ba** |
| Calabria | ITF6 | **Cal** |
| Campania | ITF3 | **Cam** |
| Emilia-Romagna | ITH5 | **ER** |
| Friuli-Venezia Giulia | ITH4 | **FVG** |
| Lazio | ITI4 | **La** |
| Liguria | ITC3 | **Li** |
| Lombardia | ITC4 | **Lo** |
| Marche | ITI3 | **Ma** |
| Molise | ITF2 | **Mo** |
| Piemonte | ITC1 | **Pi** |
| Puglia | ITF4 | **Pu** |
| Sardegna | ITG2 | **Sa** |
| Sicilia | ITG1 | **Si** |
| Toscana | ITI1 | **To** |
| Trentino- | ITH10 | **Tn** |
| Alto Adige | ITH20 | **AA** |
| Umbria | ITI2 | **Um** |
| Valle d'Aosta | ITC2 | **Va** |
| Veneto | ITH3 | **Ve** |

Figure S 2: the following map, taken from Pilli et al., 2025, reports the 19 administrative regions considered within the present study, plus the autonomous provinces of Trentino and Alto Adige (see the table above). The figure also highlights Mediterranean regions and regions affected by Vaia windstorm.

Table S 2: CBM codes attributed to the classifier Forest Type (FT), and corresponding category as defined by INFC 2005. The table also reports the broadleaves (Broad) and coniferous (Con) species’ group attributed to each FT.

| **CBM FT** | **INFC category** | **CBM species group** | **INFC area (kha)** | **Share of forest area** |
| --- | --- | --- | --- | --- |
| **AA** | Silver fir | **Con** | 68.4 | 1% |
| **BP** | Broadleaves plantations | **Broad** | 40.1 | 0% |
| **CP** | Coniferous plantations | **Con** | 15 | 0% |
| **CS** | Chestnut | **Broad** | 788.4 | 9% |
| **FS** | Beech | **Broad** | 1035.1 | 12% |
| **LD** | Larch & mountain pines | **Con** | 382.4 | 4% |
| **OB** | Other broadleaves | **Broad** | 994.8 | 11% |
| **OC** | Other conifers | **Con** | 63.4 | 1% |
| **Oca** | Hornbeam | **Con** | 852.2 | 10% |
| **OE** | Other evergreen | **Broad** | 84.7 | 1% |
| **PA** | Norway spruce | **Con** | 586.1 | 7% |
| **PM** | Mediterranean pines | **Con** | 226.1 | 3% |
| **PN** | Black pine | **Con** | 236.5 | 3% |
| **PP** | Poplar plantations | **Broad** | 66.3 | 1% |
| **PS** | Scots pine | **Con** | 151.7 | 2% |
| **QC** | Turkey oak | **Broad** | 1010.1 | 12% |
| **QI** | Evergreen oak | **Broad** | 620.3 | 7% |
| **QR** | Common oak | **Broad** | 1084.2 | 12% |
| **QS** | Cork oak | **Broad** | 168.6 | 2% |
| **RF** | Riparian forests | **Broad** | 229 | 3% |
| Total Italy (excluding temporary unstocked area) | |  | 8703.4 |  |

Table S 3: regional distribution of the total forest area between forest area available (FAWS) and not available (FNAWS) for wood supply.

| **Region** | **Share** | |
| --- | --- | --- |
|  | **FAWS** | **FNAWS** |
| **AA** | 90% | 10% |
| **Ab** | 85% | 15% |
| **Ba** | 95% | 5% |
| **Cal** | 90% | 10% |
| **Cam** | 86% | 14% |
| **ER** | 91% | 9% |
| **FVG** | 61% | 39% |
| **La** | 91% | 9% |
| **Li** | 95% | 5% |
| **Lo** | 89% | 11% |
| **Ma** | 98% | 2% |
| **Mo** | 97% | 3% |
| **Pi** | 92% | 8% |
| **Pu** | 99% | 1% |
| **Sa** | 92% | 8% |
| **Si** | 92% | 8% |
| **Tn** | 72% | 28% |
| **To** | 96% | 4% |
| **Um** | 97% | 3% |
| **Va** | 66% | 34% |
| **Ve** | 91% | 9% |
| **Total Italy** | **90%** | **10%** |

### Appendix B: growth curves assessment

Growth curves applied to even-aged stands were derived from volume and increment data reported by INFC 2005 for even-aged high forests, coppices, and plantations (Gasparini et al., 2011). Forest stands classified as special MTs were considered as uneven-aged. Volume data were used to derive the initialization (yield table) YT and increment data were used for deriving the current YT applied during the model run. In both cases, we started by using, if available, volume and increment data distributed by FT, MT (distinguishing high forests and coppices^[1]^), regions and age classes. Original volume and increment data were preliminarily corrected, and they were interpolated through a combined exponential and power function (Sit, 1994), using the SAS^©^ PROC NLIN procedure.

When the number of records (i.e. volume or increment data) reported by INFC for each FT, MT and region was considered as representative of different age classes (i.e., INFC 2005 reports at least 4 age classes for each FT, MT, and region), we defined species-specific growth curves, parametrized at regional level.

When the number of records reported by INFC was not representative or the growth curves derived by the previous analysis were not consistent with original input data (each growth curve was further validated by an expert assessment) or PROC NLIN failed to converge, we derived a second, generic, dataset only distinguished by FT and MT. These generic growth curves were further scaled at regional level by comparing, for each FT and MT, the average volume and increment reported by INFC for each region, FT and MT, and within the same range of age classes reported by INFC, with the corresponding average volume and increment derived by the generic growth curves dataset. In this way, also the volume and increment data derived by the generic growth curves were rescaled at regional level according to the specific values reported by INFC for each FT and MT.

Volume growth curves were directly used as initialization YTs, deriving the theoretical volume evolution up to an age class of 210 years. Increment growth curves were preliminarily converted to current yield tables by deriving the cumulate increment volume associated to each age class, until a volume age class corresponding to 210 years.

For the final initialization library, species-specific growth curves for even-aged forests were assigned to 15% of high forest stands and 50% of coppices stands. Species-specific growth curves were assigned to 13% of high forest stands and 48% of coppices stands. All growth curves were also validated through a final expert assessment.

For plantations, including poplars, broadleaves, and coniferous plantations, the INFC 2005 reports a generic data series of volume and increment data by age classes, distributed by regions, but not distinguished by forest types. We considered these data as mostly referred to broadleaves and we applied the corresponding correction factors. Because data available for single regions were not sufficient to derive specific growth curves at regional level, we first derived a generic volume and increment growth curve, and then we scaled the generic growth curve at regional level and by forest type, according to the same approach applied before.

Growth curves applied to uneven-aged stands were derived from NFI plot level data collected within the Third Italian NFI (INFC, 2015). We selected from the original database, including 6,993 plots, the following parameters:

- *Vapv_m*: volume per hectare of living trees (stem + large branches) in m^3^ ha^-1^ yr^-1^.
- *ICVapv_m*: current annual volume increment per hectare of living trees in m^3^ ha^-1^ yr^-1^.
- *cod_istat*: administrative region code, to be associated to CBM regions.
- *codcfor*: forest category code, to be associated to CBM forest types.

Volume and increment data were preliminarily corrected using the same CF applied to even-aged stands. Growth curves were defined at FT and regional level through the following steps:

1. We preliminarily excluded from the original database all the groups of FTs and regions with a number of plots < 10.
2. We derived the percentage annual increment (ICP) associated to each plot, calculated as follows:

$ICP=\frac{ICVapv\_m}{Vapv\_m}*100$	Eq. (1)	4

3. Possible outliers, defined as observations with a distance greater than 3 interquartile ranges from the median of each group (SAS Institute Inc., 1990), were excluded from the database using SAS^©^ PROC UNIVARIATE procedure. For this purpose, we first identified and excluded volume’s outliers (*Vapv_m*), then increment’s outliers (*ICVapv_m*) and, finally, we excluded the percentage annual increment’s outliers (*ICP*). The remaining observations define the *Clean_database* considered for the following steps.
4. For each FT and region, we derived from the *Clean_database* a quadratic regression model defined by the following equation:

$y=\alpha x+ \beta x^{2}$	Eq. (2)

where y = *ICVapv_m*, x = *Vapv_m* and parameters *α* and *β* were estimated using the SAS^©^ PROC REG procedure. An example of the regression model application for the forest type LD and region Tn is reported on Figure S 3.

Figure S 3: volume and increment per ha reported by plot level data (blue points) and estimated by the regression model reported on eq. 5 (red points) for the forest type LD (Larix decidua) and region Tn (Trento province).

For each equation, the regression model accuracy was evaluated through the adjusted R^2^ parameter calculated by the PROC REG procedure (e.g., for the example reported on Figure S 3, *R^2^* = 0.89). All equations with an error degree of freedom < 10 were excluded from the following steps.

5. We identified the minimum (*V_min_*) and maximum (*V_max_*) volume per ha reported within the *Clean_database* for each FT and region and we preliminarily distributed the corresponding volume range between 21 volume classes, with a constant volume range (*V_range_*) equal to:

$V_{range}=\frac{(V_{max}- V_{min})}{20}$	Eq. (3)

6. We estimated the annual increment (*i_Cl_*) associated to each volume class according to the regression models defined, for each FT and region, with Eq. (2) (see for example Figure S 4).

Figure S 4: example of annual increment associated to each volume class (CL_1, CL_2, etc,) derived by the regression model defined by eq. (5) for the forest type LD and region Tn.

The resulting increment values were used to derive, for each FT and region, species-specific uneven-aged growth curves including 21 volume classes (*cl*), with the volume of each class *n* ($V_{C}^{n}$) defined as follow (see Figure S 5):

$V_{Cl}^{n}=V_{Cl}^{n-1}+10*{cai}_{Cl}^{n-1}$	Eq. (4)

Where *cai_CL_* is the annual increment estimated on the previous step.

Figure S 5: example of cumulated volume per ha (reported on the left y-axis) derived from the current annual increment values (reported on the right y-axis) associated to each volume class (reported on the x-axis) defined for the forest type LD and region Tn. Red points highlight the volume and increment values derived by INFC input data (preliminarily corrected), for uneven-aged forest stands within the same forest type and region.

7. Regression models defined in step (4) do not include all the uneven aged forest stands reported by INFC input data, because some stands were excluded from the analysis (e.g., because considered as outliers or with a number of plots < 10). For this reason, we defined, for each FT, a second set of generic growth curves, to be applied only where species-specific growth curves were missing. This second set of growth curves was defined, for each FT and excluding extreme volume data^[2]^, by the mean volume derived, for each age class, by the species-specific growth curves defined on step (6).
8. Combining the species-specific growth curves defined by Eq. (4) with the generic growth curves defined on step 7 we provided, for all FT^[3]^ and regions considered within the inventory table, a set of growth curves suited for uneven-aged forest stands. These curves can be directly used both as initialization yield tables and current yield tables.

When uneven-aged YTs are implemented within the CBM modelling framework, each volume class was considered as equivalent to an age class and the area assigned to each uneven-aged FT and region, was allocated within a unique “volume class”, following the same principle described by Pilli et al., 2013. To correctly simulate the initial standing volume at the beginning of the model run (time step 0), we assigned to each uneven-aged stand the volume-class corresponding to the standing volume reported by the input data used for CBM initialization (i.e., the standing volume reported by INFC 2005). For this purpose, for each FT and region, each uneven-aged stand reported within the CBM inventory input data was first associated to the volume reported by the corresponding uneven-aged growth curve, and then to the equivalent volume-class, assumed, within the CBM modelling framework, as a "virtual" age class (see the example reported on Figure S 5). Therefore, the initial age classes assigned to uneven-aged stands within the CBM inventory input data vary by region and FT, depending on the corresponding growth curve. The same assumptions were also applied to the special management type assigned to the forest types CS and QS, where no age class distribution is reported by INFC data.

**References**

Gasparini P, Tabacchi G (Eds.). L’Inventario Nazionale delle Foreste e dei serbatoi forestali di Carbonio - INFC 2005. Secondo inventario forestale nazionale italiano. Metodi e risultati. Ministero delle Politiche Agricole, Alimentari e Forestali, Corpo Forestale dello Stato; Consiglio per la Ricerca e la Sperimentazione in Agricoltura, Unità di Ricerca per il Monitoraggio e la Pianificazione Forestale. Edagricole-Il Sole 24 ore, Milano, 2011.

Pilli, R., Grassi, G., Kurz, W. A., Smyth, C. E., & Blujdea, V., 2013. Application of the CBM-CFS3 model to estimate Italy's forest carbon budget, 1995–2020. *Ecological Modelling*, *266*, 144-171.

Sit, V., 1994. Catalog of curves for curve fitting. In: Biometrics Information Handbook Series 4. Ministry of Forest, Victoria, British Columbia (Canada).

### Appendix C: biomass wood density assessment and allometric equation selection

Figure S 6: The following figure compares the biomass wood density expected by literature and provided by the model output, according to the default EU AIDB (Pilli et al., 2018) and with the new equations selected within the present study.

Table S 4: new association between Boudewyn’s allometric equations and Italian Forest Types.

| **ClassifierValueID** | **AIDB Code** | **Canadian name of the selected species (Boudewyn, 2007)** |
| --- | --- | --- |
| AA | PA_IT | Red pine |
| BP | QC_IT | Largetooth aspen |
| CP | OC_IT | Red pine |
| CS | Oca_IT | Black spruce |
| FS | QS_IT | White elm |
| LD | PA_IT | Red pine |
| OB | QR_IT | Basswood |
| OC | OC_IT | Red pine |
| Oca | QR_IT | Basswood |
| OE | QS_IT | White elm |
| PA | PA_IT | Red pine |
| PM | PM_IT | Red pine |
| PN | PN_IT | Red pine |
| PP | AA_IT | White spruce |
| PS | PS_IT | Red pine |
| QC | QS_IT | White elm |
| QI | QI_IT | White elm |
| QR | QS_IT | White elm |
| QS | QS_IT | White elm |
| RF | RF_IT | Red pine |

**References**

Pilli, R., Kull, S. J., Blujdea, V. N., & Grassi, G., 2018. The carbon Budget model of the Canadian forest sector (CBM-CFS3): customization of the archive index database for European Union countries. *Annals of forest science*, *75*(3), 1-7.

### Appendix D: distribution of harvest

Table S 5: Total fellings, as reported by Pilli et al., 2025, and total removals, as considered within the present study assuming a constant correction factor equal to 0.85. All values are reported as volume, in m3 103 over bark.

| **Year** | **Tot Fellings** | **Removals = Fellings * 0.85** |
| --- | --- | --- |
| 2005 | 13,701 | 11,646 |
| 2006 | 16,390 | 13,931 |
| 2007 | 15,663 | 13,314 |
| 2008 | 12,583 | 10,696 |
| 2009 | 11,807 | 10,036 |
| 2010 | 12,467 | 10,597 |
| 2011 | 12,786 | 10,868 |
| 2012 | 13,486 | 11,463 |
| 2013 | 11,161 | 9,487 |
| 2014 | 10,450 | 8,882 |
| 2015 | 12,151 | 10,328 |
| 2016 | 11,762 | 9,998 |
| 2017 | 11,131 | 9,461 |
| 2018 | 11,013 | 9,361 |
| 2019 | 11,638 | 9,892 |
| 2020 | 12,624 | 10,731 |
| 2021 | 11,222 | 9,539 |
| 2022 | 13,313 | 11,316 |
| 2023 | 13,143 | 11,171 |

Total removals scaled at regional level were further distributed between various FT, MT, MS and disturbance events, based on the following steps:

1. The annual amount of harvest (*H_y_*) was distributed between coniferous (*C_y_*) and broadleaves (*B_y_*) species according to the relative share of coniferous (*FAO_C_*) and non-coniferous (*FAO_NC_*) roundwood removals reported, for each year (*y*), by FAOSTAT data series:

$C_{y}=H_{y}*{FAO}_{C}$	Eq. (8)

$B_{y}=H_{y}*{FAO}_{NC}$	Eq. (9)

2. We estimated the share of harvest assigned to each region (*r*) in 2005 ($P_{2005}^{r}$) and 2015 ($P_{2015}^{r}$) based on the total fellings reported by INFC 2005 and 2015 (see tab. 1.11.1, INFC data, excluding plantations). The harvest distribution over the intermediate period 2006 – 2014 was inferred as linear interpolation between 2005 and 2015. Between 2016 and 2018 we applied the same share reported by INFC for 2015. Since 2019, but the share of harvest was modified according to the specific assumptions linked to salvage logging activities carried out after the windstorm occurred in autumn 2018.
3. By comparing the total fellings reported by INFC 2005 and 2015 with the amount of fellings assigned to the total forest area, we estimated the share of harvest provided by plantations in 2005 and 2015 (*PL_2005_* and *PL_2015_*). In this case, the data series was not scaled at regional level due to the high uncertainties of data collected by INFC for this forest category. The distribution within the period 2006 – 2014 was inferred as linear interpolation between 2005 and 2015. Since 2016 onward, we applied the same share reported by INFC for 2015.
4. By combining the amount of harvest assigned to broadleaves and coniferous species as determined on point 1 (*C_y_* and *B_y_*), with the annual share of fellings was assigned to each region ($P_{y}^{r}$). We estimated the absolute amount of harvest expected for each species’ group (${Hb}_{y}^{r}$and ${Hc}_{y}^{r}$ for coniferous and broadleaves species, respectively), for each year (*y*) and region (*r*), excluding the amount of harvest assigned to plantations for the same year (*PL_y_*):

${Hb}_{y}^{r}=B_{y}*P_{y}^{r}*(1-{PL}_{y})$	Eq. (10)

${Hc}_{y}^{r}=C_{y}*P_{y}^{r}*(1-{PL}_{y})$	Eq. (11)

5. Based on the forest area available for wood supply (i.e., excluding FNAWS) as defined in the inventory table, we estimated the share of area assigned to each FT, MT and MS, within each region and species’ group (*Prop_FT_*__MT_MS_), excluding poplar, broadleaves and coniferous plantations as far as the “special” MT.
6. The amount of harvest defined on point 4 was proportionally distributed between each FT, MT and MS according to the share defined on point 5.
7. We defined a preliminary dataset including about 100 potential silvicultural treatments with different harvest intensities, available for each combination of FT, MT and MS within different age class intervals.
8. The amount of harvest assigned to each region/FT/MT/MS (see point 6), was further distributed between different disturbance events (i.e., essentially thinnings and final cuts for even-aged forest stands^[4]^), according to the relative proportion of area available for harvest at the beginning of the model run (i.e., time step 0, as reported within the inventory tables), under (for thinnings disturbance events ) and over (for final-cuts) the minimum rotation length associated to each FT.
9. The amount of harvest assigned to each region/FT/MT/MS/disturbance event (see step 8), was further converted to merchantable carbon (expected as input within the disturbance event table) by using species-specific WD values, taking into account also of the extra amount of harvest associated, for each disturbance event, to removals of stem snags and other wood components (i.e. branches, as quantified within the disturbance matrix associated to each disturbance event). These amounts were computed through a preliminary model run.
10. Harvest assigned to plantations was computed using a similar approach to provide a preliminary disturbance event table, including the total amount of harvest (in t of C) expected within the calibration period, distributed between various region/FT/MT/MS/disturbance events. These data were used for a preliminary model run.
11. The output of the preliminary model run was further compared with the amount of harvest expected by the input data defined in step 10 and, if needed, further corrected for each time step, redistributing to other groups (i.e., coniferous and broadleaves groups) the harvest not provided by some region/FT/MT/MS/disturbance events. The resulting database was finally used as disturbance event table for the calibration period.

**References**

Pilli R, Runge A, Chirici G, Vangi E, Collalti A, Herold M. Integrated analysis of harvest statistics provided by remote sensing, National Forest Inventories and administrative survey systems: an example from Italy. *International Journal Applied Earth Observation and Geoinformation*, 2025;*144*,104871

### Appendix E: analysis of ancillary data reported by literature for the area affected by Vaia windstorm

The forest area affected by Vaia windstorm, which occurred in autumn 2018 (ca. 52,489 ha), was distributed between five administrative regions over the period 2019 and 2022 and about 6 million m^3^ of wood were removed through salvage logging, mostly concentrated in the Veneto and Trento Alto-Adige regions (Table S 6).

Table S 6: the following table summarizes data reported in the literature on the volume and area affected by Vaia windstorm, and the volume removed by salvage logging activities carried out between 2019 and 2022 within the regions affected by Vaia.

| Data input | | | | |
| --- | --- | --- | --- | --- |
| Region/Autonomous province | Vol. damaged  (m^3^ 10^3^) | Area  (ha) | Salvage Logging  (m^3^ 10^3^)  (% on vol. damaged) | Data source |
| Trento province | 4,058 | 19,545 | 2,480* (60%) | [1] |
| Alto-Adige province | 1,536 | > 5,000 | 1,627** (106%) | [3] |
| Trento+Alto Adige | 5,594 | > 24,545 | 4,107 (73%) | - |
| Veneto | 2,800 | ≈ 20,000 | 1,844*** (65%) | [2] - [4] |
| Friuli-Venezia-Giulia | 800 | 3,340 | n.r. | [2] |
| Lombardia | 300 | 4,604 | 255 | [2] - [5] |
| Piemonte | 1 | -- | n.r. | [2] |
| Total | 9,495 | > 52,489 | > 5,951 (≈ 62%) | - |
| [1] AA. VV., 2020 - Piano d’azione Vaia in Trentino. L’evento, gli interventi, i risultati. 72 pp. In: Sherwood 248 Supplemento 2 | | | | |
| [2] GeoLab, Florence University, as reported by Cozzarini (2018), and other data collected at local level | | | | |
| [3] Provincia Autonoma di Bolzano, Ripartizione Foreste. Vaia 2018. Report finale. Dicembre 2020 | | | | |
| [4] data collected at local level | | | | |
| [5] Celona et al., 2023. Rapporto sullo stato delle foreste in Lombardia 2022. ERSAF, Regione Lombardia | | | | |
| * 2019 – June 2020  ** 2019 - 2020  *** 2019 – 2022  n.r.: not reported/not available | | | | |

To simulate the effects of Vaia windstorm and Bark Beetle outbreaks and of salvage logging activities carried out between 2019 and 2023, we applied the following disturbance events:

| **Disturbance event** | **Share of biomass moved to other pool** | **Applied to** |
| --- | --- | --- |
| Damage class I | 15% from merch. biomass to DOM | 2018 & 2020/21 |
| Damage class II | 40% from merch. biomass to DOM | 2018 & 2020/21 |
| Damage class III | 70% from merch. biomass to DOM | 2018 & 2020/21 |
| Damage class IV | 95% from merch. biomass to DOM | 2018 & 2020/21 |
| Salvage logging after windstorm/Bark beetle outbreaks | 90% from snag stem (DOM) to products pool | 2019 – 2023 |

Input data reported by literature were compared with the model output, as reported by the following table:

| Region/Autonomous province | CBM Model output | | | | |
| --- | --- | --- | --- | --- | --- |
|  | Merch. vol. damaged  (m^3^ 10^3^) | Area  (ha) | Salv. Log. 2019^ǂ^  (m^3^ 10^3^) | Salv. Log. 2020^ǂ^  (m^3^ 10^3^) | Salv. Log. 2019/20^ǂ^  (m^3^ 10^3^ ) |
| Trento province | 3,946 | 17,375 | 1,503 | 997 | 2,500 |
| Alto-Adige province | 1,687 | 5,414 | 1,267 | 244 | 1,511 |
| Trento+Alto Adige | 5,633 | 22,789 | 2,770 | 1,241 | 3,611 |
| Veneto | 2,749 | 17,857 | 777 | 477 | 1,254 |
| Friuli-Venezia-Giulia | 797 | 3,447 | 242 | 164 | 406 |
| Lombardia | 431 | 2,547 | 157 | 108 | 265 |
| Piemonte | 1 | 6 | 0.4 | 0.5 | 0.9 |
| Total | 9,611 | 46,648 | 3,946 | 1,990 |  |
| ^ǂ^ including non-merchantable components | | | | | |

**References**

AA. VV., 2020 - Piano d’azione Vaia in Trentino. L’evento, gli interventi, i risultati. 72 pp. In: Sherwood 248 Supplemento 2

Celona, F., Guglini, M., Craveri, L., Bazzoli, M., Siena, F., Cornaggia, N. (2023). Rapporto sullo stato delle foreste in Lombardia 2022. ERSAF, Regione Lombardia

Cozzarini E (2018) Tempesta Vaia, i danni per le foreste del Nordest. La nuova Ecologia.

Provincia Autonoma di Bolzano, Ripartizione Foreste. Vaia 2018. Report finale. Dicembre 2020

### Appendix F: supplementary results data

### F1. Merchantable volume and aboveground biomass stock estimated by CBM and reported by NFI data, scaled at national and forest type level

Figure S 7: Comparison between the over bark (o.b) merchantable volume (including branches) by Forest Type estimated by CBM for 2005 and 2015 and the corresponding merchantable volume, reported by INFC 2005 and 2015. Bars associated to INFC values correspond to the percentage error reported by INFC data for each Forest type.

Figure S 8: Comparison between the aboveground biomass stock by Forest Type estimated by CBM for 2005 and 2015 and the corresponding biomass stock reported by INFC 2005 and 2015. Bars associated to INFC values correspond to the percentage error reported by INFC data for each Forest Type. All values refer to Mg dry biomass per ha.

### F2. Comparison between CBM and INFC DOM components

The carbon stored in snag stems and medium DOM pools modelled by CBM can be compared with NFI pools by converting mass to volume, using country-specific basic wood density values (Morelli et al., 2007, Paletto and Tosi, 2010). To achieve this, we estimated snag stem volumes using wood density values assuming a first decay class (“recently dead trees”),as reported by Morelli et al. (2007) and Paletto and Tosi (2010), and we assigned the medium DOM sub-pool using the wood density assigned to the medium decay class (fragments of bark only) as reported by the same authors.

**References**

Morelli S, Paletto A, Tosi V. Il legno morto dei boschi: indagine sulla densità basale del legno di alcune specie del Trentino. Forest@, 2007;4(4),395-406.

Paletto A & Tosi V. Deadwood density variation with decay class in seven tree species of the Italian Alps. *Scandinavian Journal of Forest Research*, 2010;*25*(2),164-173.

### F3. Net stock change derived from INFC 2015

Annualization of derived stock change estimates is challenging. Indeed, on one hand, the annual volume increment provided by INFC 2015 is based on the annual increment of stem radius derived from the average of the five outermost rings (Gasparini et al., 2022), and, on the other hand, the amount of fellings is mostly attributed to a period spanning between 2017 and 2019 (Pilli et al., 2025). Therefore, we can assume that the average C stock change inferred from INFC 2015 can be broadly referred to the period 2015-2017.

The following table reports the net C stock change as derived by INFC 2015 taking into account the gross annual increment (GAI) and the amount of fellings estimated by NFI and the corresponding confidence intervals, and the annual natural losses as derived, for the Italian NFI, from Avitabile et al. 2024.

| INFC 2015 | Unit | Note | INFC data | INFC % error | Confidence interval | |
| --- | --- | --- | --- | --- | --- | --- |
| GAI | Mg C | see Fig 12.9, INFC 2015 | 13,548,919 | ± 1% | 13,413,430 | 13,684,408 |
| FELLINGS | Mg C | see Fig 12.9, INFC 2015 | 3,164,519 | ± 15% | 2,677,183 | 3,651,855 |
| ANNUAL NATURAL LOSSES | Mg C | 14% of GAI, Avitabile et al. 2024 | 1,896,849 | not available | 1,896,849 | 1,896,849 |
| NET STOCK CHANGE | Mg C | Sum | 8,487,551 | Sum | 8,839,398 | 8,135,705 |
|  | Gg CO2 | Sum *-44/12*1/1000 | -31,121 |  | -32,411 | -29,831 |

**References**

Avitabile, V., Pilli, R., Migliavacca, M., Duveiller, G., Camia, A., Blujdea, V., ... & Mubareka, S. (2024). Harmonised statistics and maps of forest biomass and increment in Europe. *Scientific data*, *11*(1), 274.

Gasparini P, Di Cosmo L, Floris A, De Laurentis D. Italian National Forest Inventory—Methods and Results of the Third Survey. Springer Open Access, 2022.

Pilli R, Runge A, Chirici G, Vangi E, Collalti A, Herold M. Integrated analysis of harvest statistics provided by remote sensing, National Forest Inventories and administrative survey systems: an example from Italy. *International Journal Applied Earth Observation and Geoinformation*, 2025;*144*,104871.

1. For growth curves, coppices were not further distinguished between different MS.
2. Extreme volume data were identified, for each FT and age class, as observations with a distance greater than 3 interquartile ranges from the median of each group.
3. Excluded poplar, broadleaves and coniferous plantations.
4. Coppices and uneven-aged stands only include one disturbance type.
